# Autism Observation Scale for Infants: Systematic Review and Meta-Analysis in Samples at Increased Likelihood of Autism Spectrum Disorders

**DOI:** 10.1007/s40489-023-00417-y

**Published:** 2024-01-18

**Authors:** Kyle Reid, Lori-Ann R. Sacrey, Lonnie Zwaigenbaum, Jessica A. Brian, Isabel M. Smith

**Affiliations:** 1 Autism Research Centre, Department of Pediatrics, Glenrose Rehabilitation Hospital and University of Alberta, Edmonton, Canada; 2 Autism Research Centre, Department of Pediatrics, Holland Bloorview Research Institute and University of Toronto, Toronto, Canada; 3 Autism Research Centre, Departments of Pediatrics, Psychology, and Neuroscience, IWK Health Centre and Dalhousie University, Halifax, Canada

**Keywords:** Autism Spectrum Disorder, Autism Observation Scale for Infants, Infant Siblings, Fragile X Syndrome, Tuberous Sclerosis Complex, Down Syndrome, Increased Likelihood

## Abstract

The Autism Observation Scale for Infants (AOSI) is being applied to non infant sibling populations. Assessment of the tool’s utility across increased likelihood (IL) populations is therefore needed. A systematic review and meta-analysis was conducted on 17 studies identified from six databases. The AOSI has been used in four IL contexts: infant siblings, infants with Fragile X Syndrome, Tuberous Sclerosis Complex, and Down Syndrome. There were three main findings: (1) five studies report classification data though no consistent approach was used; (2) group differences between IL-ASD, IL non-ASD, and controls started at 12-months; and (3) large effect sizes between IL-ASD and control samples was identified. Utility of the AOSI to identify early signs of ASD in IL populations was demonstrated.

**Supplementary Information:**

The online version contains supplementary material available at 10.1007/s40489-023-00417-y.

Autism Spectrum Disorder (ASD) is a neurodevelopmental condition characterized by differences or impairments in social-communication and the presence of restricted interests, repetitive behaviours, and/or atypical responses to sensory input (American Psychiatric Association 2022). The current community prevalence rate of ASD as reported in the United States by the Centers for Disease Control and Prevention (CDC) is 1 in every 36 children by age 8 (Maenner et al., 2023). There are some populations who are at an increased likelihood (IL) for developing ASD due to environmental or genetic factors such as increasing paternal age, children with premature birth, fragile X syndrome (FXS), and tuberous sclerosis complex (TSC; Abbeduto et al., 2014; Agrawal et al., 2018; Capal et al., 2017; Hultman et al., 2011). Because ASD is characterized by highly complex and variable phenotypic presentation, it is important to assess the utility of any measure attempting to investigate early features of ASD.

The increasing recognition of the benefits of early intervention for children on the autism spectrum (Fuller & Kaiser, 2020, Towle et al., 2020, Dawson et al., 2010, Bonis, 2016, Pickles et al., 2016, Noyes-Grosser et al., 2018) highlight the need of early assessments like the AOSI which can provide behavioural data that supports access to early intervention and diagnostic services (Gardner et al., 2013, Fuller & Kaiser, 2020, Towle et al., 2020). It is important for primary care practitioners to provide referrals to specialists and early intervention services (Zwaigenbaum et al., 2015). Given that gold-standard ASD diagnoses are very stable (94% of infant siblings of children on the autism spectrum followed from ages 3 to 9 years retained a diagnosis in Brian et al., 2016’s study), tools that aid in early identification of ASD have potential utility to facilitate access to early intervention services.

The Autism Observation Scale for Infants (AOSI) is a brief, 19-item observational measure that was initially designed to characterize early behavioural signs of ASD between 6 and 18 months in a familial cohort of infants at increased likelihood of the disorder (i.e., are infant siblings of children diagnosed with ASD; Bryson et al., 2008). The AOSI assesses multiple overlapping constructs that characterize prodromal ASD (e.g., social communication, emotional regulation, atypical sensory-motor behaviours, repetitive behaviours, etc.) within an interactive, play-based context in which behaviour can be systematically elicited by trained examiners (Bryson et al., 2008). A child can be scored using the AOSI in two different ways: (1) by calculating their AOSI Total Score (a summed score of items 1 to 18 on the scale; values ranging between 0 and 38), or (2) by calculating the number of AOSI Risk Markers they exhibit (a tally of items 1 to 18 where the participant score at least a 1 or higher with values ranging between 0 and 16; Bryson et al., 2008; Zwaigenbaum et al., 2005). Though the AOSI has been validated in IL infant siblings, research groups are starting to assess the tool for use in identifying early signs of ASD in other populations of infants at IL for ASD including infants who were born premature, or who have underlying genetic or neurological conditions such as Down Syndrome (DS; Hahn et al., 2020; Sanderson, 2016). Yet, early signs of ASD may be expressed differently across these populations. The purpose of this systematic review and meta-analysis is to provide an in-depth examination of research assessing the individual classification properties and group differences of the AOSI across different IL groups from 6–18 months to examine if early signs of ASD present differently across different IL populations.

## Methods

### Search Strategy

A systematic review was completed following the Preferred Reporting Items for Systematic Reviews and Meta Analyses (PRISMA; Page et al., 2021) checklist. Searches were performed on July 4th, 2022, in six databases: CINAHL, EMBASE-OVID, ERIC, JSTOR, PubMed, and Web of Science. Search terms and strategies were refined following discussion between two reviewers (K.R. and L.S.) using the terms “Autism Observation Scale for Infants,” “AOSI,” and "autism", “autism spectrum disorder,” and “autistic disorder.” No published search filters were used. Because the AOSI was first published in 2005, date limits for the primary search were set to identify articles published between January 1^st^, 2005, and July 4^th^, 2022. Although no language limits were used to allow for capture of any non-English publications (as the AOSI has been translated into other languages, such as Hebrew; Ben-Sasson & Carter, 2012), no non-English studies were identified. Primary database searches identified 453 articles. Grey literature databases (opengrey.eu, worldcat.org, greylit.org) were surveyed using identical search terms used in primary database searches to identify relevant unpublished data and identified 27 articles. The same search terms were employed in the primary and grey literature searches. One article, Zwaigenbaum et al., 2005, was manually imported for primary screening as study authors knew it was the first paper published on the AOSI but was not captured in either the primary or grey literature search. In preparation for publication, a second search was conducted on July 26^th^, 2023, using the same search strategy but limited to articles published between July 4^th^, 2022, and July 26^th^, 2023. Though an additional 32 articles were identified, none met inclusion criteria. IL groups were not pre-specified for either search to be as inclusive as possible and not potentially exclude articles from IL populations unknown to study authors. In total, 513 articles were imported into Covidence (covidence.org) for review. Following de-duplication, 383 articles were identified for further screening. The complete search strategy as run and PRISMA checklist can be found in Supplementary Files 1 and 2, respectively. Though no PROSPERO protocol for this review was registered, the PROSPERO database was searched to ensure no other similar review had been registered or conducted prior to this study. As of August 4th, 2023, while one protocol was identified that used the AOSI (CRD42020158688), the AOSI was used as an outcome measure in a systematic review of ASD-related interventions in the first 2 years of life and thusly does not conflict with this review focusing on AOSI classification properties.

### Screening for Inclusion and Exclusion Criteria

**Fig. 1:**
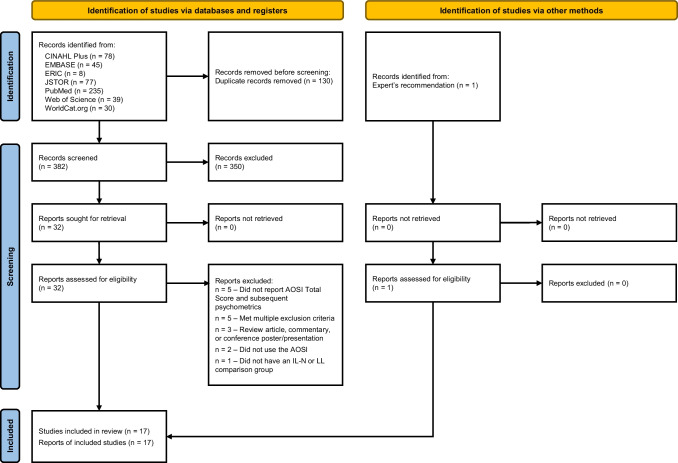
Systematic review strategy using the 2020 PRISMA method (Page et al., 2021)

To be included in this review, a paper (1) used the AOSI in a population of IL infants characterized by a specific factor known to be associated with increased likelihood of ASD diagnosis (e.g., infant siblings of a child with ASD, infants with FSX, TSC, or DS) and a sample of control infants (low likelihood [LL] or IL infants not diagnosed with ASD), (2) either reported at least one AOSI cut point and its corresponding sensitivity and specificity or compared AOSI Total Scores between two or more groups, and (3) included original data. A paper was excluded from analysis if it (1) did not use the AOSI, (2) did not include AOSI Total Scores, number of AOSI Risk Markers, or sensitivity and specificity data, (3) lacked a comparison group (IL-not diagnosed/IL-N; LL controls), or (4) was a review article, commentary, conference abstract, or conference presentation. Titles and abstracts of 354 articles were screened using the reported inclusion and exclusion criteria in Covidence by two independent reviewers (K.R. and L.A.) to identify the studies meriting full-text review. Both reviewers assessed the 33 articles meriting full-text review and had 97% agreement for studies meeting inclusion criteria. The one disagreement was resolved by consensus following discussion between reviewers. In total, 17 articles were selected for full-text extraction, with nine included in meta-analyses. The PRISMA flow diagram for this review, including reasons for exclusion at the full-text review level, is described in Fig. 1.

### Assessment of Quality and Risk of Bias

Authors of the *Cochrane Handbook for Systematic Reviews of Interventions* recommend focusing on assessing risk of bias over methodological quality (Stang et al., 2018). For this reason, study quality, methodology, and potential sources of bias were assessed using a composite form generated using items from the National Institute of Health’s Quality Assessment Tool for Observational Cohort and Cross-Sectional Studies (National Heart, Lung, and Blood Institute, 2021), the Joanna Briggs Institute’s Checklist for Systematic Reviews and Research Syntheses (Moola et al., 2017), the Critical Appraisal Skills Programme Checklist for Cohort Studies (CASP 2018), and the Scottish Intercollegiate Guidelines Network’s Methodology Checklist 3 for Cohort Studies (Scottish Intercollegiate Guidelines Network 2016). K.R and L.S. generated the table assessing for potential sources of bias in the included studies. Both authors independently scored each of the 17 studies by each category in the risk of bias table before comparing scores. Any disagreements were resolved via consensus. One intent behind this review and meta-analysis was to highlight potential sources of bias that may warrant further investigation or consideration as it relates to study quality and validity, as well as to facilitate a discussion of the generalizability of results.

### Data Extraction

Two primary reviewers (K.R. and L.S.) developed a standardized data extraction form. Extracted demographic information included sample size, the IL population being examined, sex ratio, ethnicity, parental age, and socioeconomic status (SES). AOSI-relevant information and potential biasing factors extracted from the studies included inclusion/exclusion criteria; chronological age at assessment; statistical method; covariates; ASD classification/diagnostic assessment; AOSI cut points, sensitivity, and specificity; group comparisons; data required to calculate effect sizes (IL/LL sample sizes, AOSI Total Score and standard deviation data); and study limitations. The data extraction form was iteratively developed to allow for flexibility and comprehensiveness (Colquhoun et al., 2014). Both primary reviewers each extracted data from a portion of the 17 included studies and cross-checked the other’s work for validation purposes.

### Statistical Analysis

Meta-analyses on AOSI Total Score were completed in Stata using the *metan* command (Sterne, 2009). AOSI meta-analyses were stratified by age based on the results of Zwaigenbaum et al., 2020 who report IL-ASD and IL-N infant sibling group differences emerge beginning at 12-months using the AOSI Total Score. Thusly, articles that included AOSI scores under 12-months were not pooled with articles that report AOSI scores at or following 12-months. Eight separate meta-analyses were conducted: (1) studies with LL controls versus IL infants later categorized with ASD at 24- or 36-months (IL-ASD) who were assessed using the AOSI between 6–10 months, (2) studies with LL controls versus IL-ASD assessed between 12–14 months, (3) studies with IL infants not diagnosed with ASD versus IL-ASD assessed between 6–10 months, with differences between the comparison group explored using the subgrouping command, (4) studies with IL infants not diagnosed with ASD (IL Non-ASD) versus IL-ASD assessed between 12–14 months, with differences between the comparison group explored using the subgrouping command, (5) studies with IL infants not diagnosed with ASD but who were classified as having a developmental delay versus IL-ASD assessed between 6–10 months, (6) studies with IL infants not diagnosed with ASD but who were later classified as having a developmental delay versus IL-ASD assessed between 12–14 months, (7) studies with IL infants with typical development (e.g., IL infants without any developmental concerns) versus IL-ASD assessed between 6–10 months, and (8) studies with IL infants with typical development versus IL-ASD assessed between 12–14 months.

Cohen’s d effect sizes (calculated using the following formula: d = M_1_ – M_2_/σ_spooled_ where σ_spooled_ = √[(σ_1_^2^ + σ_2_^2^)/2]) and standard errors were computed for each study (for which data were available) and used in the meta-analyses, with d = 0.2 – 0.49 = small effect, d = 0.5 – 0.79 = medium effect, and d ≥ 0.8 = large effect (Cohen, 1992). Heterogeneity was examined using confidence intervals (CI), the* I*^*2*^ statistic, and forest plots. The *I*^*2*^ statistic, which ranges from 0 to 100%, is a measure of the variability in effect estimates resulting from heterogeneity between studies rather than chance (e.g., sampling error; Higgins et al., 2019). Statistical heterogeneity can be considered unimportant between 0–40%, moderate between 30–60%, substantial between 50–90%, and considerable between 75–100% (Higgins et al., 2019). Preliminary analyses suggested our meta-analyses had *I*^*2*^ statistics > 50%, thus we adopted random effects models for our meta-analyses. Funnel plot, trim and fill analyses, and Egger’s tests for small study effects were completed using the *metafunnel*, *metatrim*, and *metabias* commands in Stata (Sterne, 2009) to investigate publication bias and heterogeneity through visual and statistical examination of the data (Egger et al., 1997).

Overall, 9 of the 17 articles were included in the meta-analyses (Capal et al., 2017, Hahn et al., 2020, Gammer et al., 2015, Estes et al., 2015, McDonald et al., 2017, Bussu et al., 2018, Zwaigenbaum et al. 2020, Zwaigenbaum et al., 2021a, 2021b, Hahn et al., 2017). The remaining 8 articles were not included as they were earlier studies from the same research groups or were conducted using the same study population (i.e., overlapping participants between studies). For studies conducted on the same infant cohort or published from the same research group, studies with the highest sample sizes were chosen for inclusion in meta-analyses. In addition, no study was included in the same meta-analysis more than once to prevent unduly weighting or biasing analyses.

### Ethics Statement

Ethics approval was not required for this study as it is a systematic review conducted on publicly accessible de-identified information. No informed consent was required as this article is a review and no individual participants have identifying information.

## Results

This systematic review examining the utility of the AOSI to identify early signs of ASD across different IL populations included 17 peer-reviewed articles. The results are organized as follows: a descriptive overview of the included articles with location, sample size, age, and participant demographics; an overview of the IL group status; an overview of study design and methodology; description of how and at what age(s) the AOSI was used; statistical analyses employed; AOSI cut points and their associated psychometric data, and risk of bias assessment.

### Study and Participant Demographics

#### Overview of Included Articles

Although no language limits were used in the search, all articles meeting inclusion criteria were published in English. The earliest article meeting criteria was published in 2005 and the most recent in 2021. The articles originated from three countries: Canada (n = 4), the United Kingdom (n = 6), and the United States (n = 7). Fifteen were longitudinal cohort studies (participants assessed at multiple time points) and two were cross-sectional. Total sample sizes ranged from N = 36 (Hahn et al., 2020) to N = 681 (Zwaigenbaum et al. 2020). IL subsamples ranged from n = 15 (FXS; Roberts et al., 2016) to n = 501 (infant Siblings; Zwaigenbaum et al. 2020). Several studies were either conducted by the same research group (the British Autism Study of Infant Siblings [BASIS; Gammer et al., 2015, Bussu et al., 2018, Gliga et al. 2015, Bedford et al., 2016, Bedford et al., 2017, Bedford et al., 2019]; the Canadian Infant Sibling Study [CISS; Zwaigenbaum et al., 2005, Zwaigenbaum et al. 2020, Zwaigenbaum et al., 2021a, 2021b, Sacrey et al., 2018) or using overlapping participants (see [Hahn et al., 2020, Hahn et al., 2017, Roberts et al., 2016] or [McDonald et al., 2017, Jeste et al., 2014]).

#### Participant Demographics

Of the 17 included studies, four assessed infants at multiple times between ages 3 and 24 months (Zwaigenbaum et al., 2005, Estes et al., 2015, Roberts et al., 2016, Gliga et al. 2015), eleven assessed infants at multiple times between 6 and 36 months (Capal et al., 2017, Gammer et al., 2015, McDonald et al., 2017, Bussu et al., 2018, Zwaigenbaum et al. 2020, Zwaigenbaum et al., 2021a, 2021b, Bedford et al., 2016, Bedford et al., 2017, Bedford et al., 2019, Sacrey et al., 2018, Jeste et al., 2014) and two assessed infants at one time point, between 7 and 18 months (Hahn et al., 2017, 2020). Detailed participant demographic data (including both ethnicity and SES) were only reported by three studies (McDonald et al., 2017, Zwaigenbaum et al. 2020, Zwaigenbaum et al., 2021a, 2021b) which consisted of study populations of middle-to-higher SES families of largely Caucasian ancestry. Three studies (Estes et al., 2015; Hahn et al., 2017, 2020) only report ethnicity data, and likewise feature largely Caucasian study populations (with two-thirds of participants or more being Caucasian). Two studies (Bedford et al., 2017; Sacrey et al., 2018) use SES or family demographic data in their analyses but do not directly report the results or descriptive statistics in their paper. The remaining nine studies reported no participant demographic data outside of the biological sex of the participant. Descriptive characteristics of included studies can be seen in Table 1.

**Table 1:** Study Characteristics

Article (by publication year)	Country	IL group	Sample size (IL)	Sample size (LL)	Sex Ratio	Participant or Family Ethnicity	Participant / Family SES
2005 Zwaigenbaum	Canada	Infant siblings	n = 65	n = 23	ns	ns	ns
2014 Jeste	United States	Infants with TSC	n = 40	ns	ns	ns	ns
2015 Estes	United States	Infant siblings	n = 210	n = 98	Sibs: 129 male, 81 female LL: 55 male, 43 female	86.4% Caucasian overall. ^δ^*	ns
2015 Gammer	United Kingdom	Infant siblings	n = 53	n = 50	Sibs: 21 male, 32 female LL: 21 males, 29 females	ns	ns
2015 Gliga	United Kingdom	Infant siblings	n = 82	n = 27	Sibs: 45 male, 37 female LL: 14 male, 13 female	ns	ns
2016 Bedford	United Kingdom	Infant siblings	n = 54	n = 48	Sibs:21 male, 33 female LL: 21 male, 29 female	ns	ns
2016 Roberts	United States	Infants with FXS, Infant siblings	FXS: n = 15 Sibs: n = 23	n = 17	FXS: 15 males Sibs: ns LL: ns	ns	ns
2017 Capal	United States	Infants with TSC	n = 79	n/a	TSC: 43 males, 36 female	ns	ns
2017 Hahn	United States	Infants with FXS, Infant siblings	FXS: n = 18 Sibs: n = 21	n = 22	FXS: 14 male, 4 female Sibs: 17 male, 4 female LL: 18 male, 4 female	73.9% Caucasian overall. ^δ^*	Overall, 51.7% of mothers had a college degree or higher overall. ^δ*^ No group difference observed between family income, race, or maternal education between IL/LL groups. Mean family income = $57,851.50 USD. *
2017 Bedford	United Kingdom	Infant siblings	n = 42	n = 37	Sibs: 15 males, 27 female LL: 15 males, 22 female	ns	Family income was used in analyses, but data was not directly reported
2017 McDonald	United States	Infants with TSC	n = 23	n = 21	TSC: 16 male, 7 female LL: 9 male, 12 female	73.8% Caucasian overall. ^δ^*	Overall, 78.1% of mothers had a 4-year college/some graduate school or an advanced or professional degree. *
2018 Bussu	United Kingdom	Infant siblings	n = 161	n = 71	Sibs: 85 male, 76 female LL: 31 males, 40 females	ns	ns
2018 Sacrey	Canada	Infant siblings	n = 188	n/a	Sibs: 111 male, 77 female	ns	Family demographics (participant’s birth order, number of children in the family, father’s and mother’s age at participant’s birth, and family SES) were used in analyses but not reported
2019 Bedford	United Kingdom	Infant siblings	n = 54^α^	n = 50^α^	Sibs: 21 male, 33 female LL: 21 male, 29 female	ns	ns
2020 Hahn	United States	Infants with DS	n = 18	n = 18	DS: 14 male, 4 female LL: 14 male, 4 female	66.6% Caucasian overall. *	ns
2020 Zwaigenbaum	Canada	Infant siblings	n = 501^α^	n = 180^α^	Sibs: 281 male, 220 female * LL: 97 male, 83 female *	Overall, 84.8% of fathers and 82.3% of mothers were Caucasian. *	51.36% of participants families had a Hollingshead Four-Factor Index between 51 and 66
2021 Zwaigenbaum	Canada	Infant Siblings	n = 500	n = 177	Sibs: 280 male, 220 female LL: 94 male, 83 female	Overall, 84.8% of fathers and 85.0% of mothers were Caucasian. *	52.07% of participants families had a Hollingshead Four-Factor Index between 51 and 66

DS = Down Syndrome, FXS = Fragile X Syndrome, IL = infants at increased likelihood of being diagnosed with ASD, LL = infants at low likelihood of being diagnosed with ASD, SES = Socioeconomic status, Sibs = Infant siblings of children on the autism spectrum, TSC = Tuberous Sclerosis Complex, USD = US Dollar

ns = not specified

^α^ = sample sizes varied depending on analysis

^*^ = calculated from data provided in the paper

δ = No delineation between parent and child ethnicity – ethnicity was presented without context as to if it was the parents or child/study participant

### Increased Likelihood Group Status

Four IL groups were assessed: (1) younger siblings of children formally diagnosed with ASD (hereafter infant siblings), (2) infants with FXS, (3) infants with TSC, and (4) infants with DS. All four populations have elevated rates of ASD diagnoses relative to the general population, with the prevalence rate of ASD in infant siblings, FXS infants, TSC infants, and DS infants reported to be as high as 20%, 50%, 40%, and 42%, respectively (Abbeduto et al., 2014; Hahn et al., 2020; Numis et al., 2011; Ozonoff et al., 2011; Szatmari et al., 2016). Infant siblings comprised part or all of the IL sample in 13 of the 17 studies (Zwaigenbaum et al., 2005, Gammer et al., 2015, Estes et al., 2015, Bussu et al., 2018, Zwaigenbaum et al. 2020, Zwaigenbaum et al., 2021a, 2021b, Hahn et al., 2017, Roberts et al., 2016, Gliga et al. 2015, Bedford et al., 2017, Bedford et al., 2019, Sacrey et al., 2018). Descriptions of how ASD diagnoses were confirmed in the probands (older siblings diagnosed with ASD), study inclusion/exclusion criteria, and reliability assessment can be found in Supplementary File 3. Three studies included infants with TSC (Capal et al., 2017; Jeste et al., 2014; McDonald et al., 2017), two included infants with FXS (Hahn et al., 2017; Roberts et al., 2016), and one included infants with DS (Hahn et al., 2020).

### Study Design and Methodology

An overview of study design, including study objectives, inclusion criteria, and exclusion criteria is provided in Table 2.

**Table 2:** Study Methodology

Article	Study objective	Inclusion criteria	Exclusion criteria	ASD diagnostic / outcome assessment
2005 Zwaigenbaum α	Characterization of behavioural manifestations of ASD in the first year of life of IL infant siblings of children on the autism spectrum	IL-Sibs: Have an older sibling formally diagnosed with ASD confirmed by the ADOS and a clinical interview using DSM-IV criteria. LL: Term gestation, birth weight > 2500 g	IL-Sibs: ns LL: No 1^st^ or 2^nd^ degree relative with ASD	24-month ADOS classification for ASD
2014 Jeste β	Defining early clinical, behavioral, and biological markers of ASD in IL infants with TSC	IL-TSC: Recruited through TSC specialty clinics, newborn nurseries, pediatrician’s offices, or the TSC alliance in the United States. LL: Recruited through IRB-approved infant databases in the greater Los Angeles and Boston area	IL-TSC: ns LL: Prematurity (< 37 weeks gestational age), birth trauma, developmental concerns, or immediate family history of ASD or intellectual disability	Diagnoses were based on the convergence of ADOS scores (at 18-, 24- and 36-months) and clinical judgement of a board-certified pediatric neurologist
2015 Estes	Compare IL-sibs diagnosed with ASD to those who are not with respect to (1) longitudinal trajectories of cognitive development and adaptive functioning from 6–24 months and cross-sectional differences at 6, 12, and 24 months, and (2) behavioural features at 6 and 12 months	IL-Sibs: Have an older sibling that met criteria for ASD on the SCQ and ADI-R, confirmed by medical records. LL: Typically developing older sibling who did not meet for ASD on the SCQ or Family Interview for Genetic Studies (FIGS) and had no first-degree relative with ASD or intellectual disability	All participants: (1) Genetic conditions/syndromes, (2) medical/neurological conditions affecting growth, development, or cognition (e.g. seizure disorders) or significant sensory impairments (vision/hearing loss), (3) birth weight < 2,000 g, gestational age < 36 weeks, significant perinatal adversity and/or exposure in utero to neurotoxins, (4) contraindications for MRI, (5) predominant home language other than English, (6) adopted children or half-siblings, (7) 1^st^ degree relative with psychosis, schizophrenia, or bipolar disorder, and (8) twins	24-month clinical best estimate diagnosis using DSM-IV-TR criteria assessing for ASD or PDDNOS. Two clinicians assigned diagnoses: one who conducted the diagnostic assessment, and the other (a clinical psychologist or psychiatrist) was naïve to previous examinations and IL status but reviewed testing results to provide an independent DSM diagnosis confirmation
2015 Gammer δ	To investigate if (1) AOSI scores differ between IL-Sibs and LL controls at 7/14-months, (2) if AOSI scores differ between IL-Sibs who diagnosed with ASD and those who are not, and (3) investigate any associations between ~ 7/14-month AOSI scores and later 24/36-month ADOS-G scores	IL-Sibs: have an older full/half sibling with a community clinical ASD diagnosis confirmed using the DAWBA and SCQ by expert clinicians LL: No 1^st^ degree relative with ASD, have at least one older full/half sibling who does not meet criteria SCQ criteria for ASD	IL-sibs: Significant conditions (e.g., FXS, TSC) LL: Full term birth (gestational age 37–42 weeks)	36-month diagnostic assessment conducted by four clinical researchers using ICD-10 criteria (childhood autism, PDD) based on 24- and 36-month ADOS and ADI-R
2015 Gliga δ	Investigate whether perceptual and social interaction atypicalities in IL-Sibs reflect co-expressed but biologically independent pathologies (measured by eye tracking of spontaneous orienting to letter targets presented among distractors) as suggested by a 'fractionable' phenotype model of autism	IL-Sibs: Have at least one older sibling with a community clinical diagnosis of ASD confirmed by an expert clinician using the DAWBA and SCQ LL: Have at least one older sibling, full-term birth, normal birth weight, and lack of ASD diagnoses in any 1^st^ degree family members (confirmed by a parent interview of family medical history)	IL-Sibs: Significant medical conditions in probands or extended family members LL: ns	24-month ADOS scores were used as the primary outcome measure for ASD symptomology
2016 Bedford δ	To assess whether sex differences are apparent in early autism markers (attention disengagement speed, gaze-following behaviour, the AOSI) or in the relationships between these early markers and later autistic traits	ns	All participants: Medical or developmental conditions	36-month consensus diagnostic assessment using ICD-10 criteria (ASD-sibs, childhood autism, atypical autism, PDD) using all available study data (AOSI, ADOS, SCQ, attention disengagement speed, gaze-following behaviour) made by experienced researchers
2016 Roberts δ	Contrast the profile of ASD symptoms in 12-month IL-Sibs, IL-FXS, and LL controls to identify (1) risk factors for ASD in infants with FXS, (2) the concordance rate of risk factors in IL-FXS vs IL-Sibs, and (3) to document potentially etiologically distinct ASD risk profiles across IL-FXS and IL-Sib groups	IL-FXS: Have a confirmed genetic report of FXS IL-Sibs: Have an older full sibling with a confirmed community clinical ASD diagnosis LL: Absence of known or suspected delays, no history or indicator of ASD	All participants: Neurological conditions or gestation < 37 weeks LL: Infants with developmental composite scores > 1 SD away from the mean	24-month ADOS-2 toddler module scores for 39/55 participants (10/15 IL-FXS, 16/23 IL-Sibs, and 13/17 LL controls)
2017 Capal	To determine early predictors of autism risk in infants with TSC to identify children in most need of accessing autism-specific interventions	IL-TSC: Between the 3 and 12 months old at study enrollment, met clinical or genetic criteria for TSC diagnosis	IL-TSC: Gestational age < 36 weeks at birth with significant perinatal complications (respiratory support, confirmed infection, intraventricular hemorrhage, cardiac compromise), had taken an investigational drug as part of another research study within 30 days prior to enrollment, were taking an mTOR inhibitor (rapamycin, sirolimus, or everolimus) at study enrollment, had a Subependymal Giant Cell Astrocytoma requiring medical or surgical treatment, had a history of epilepsy surgery, or had any contraindications to completion of study procedures such as MRI	24-month classification of ASD based on ADOS-2 classification
2017 Hahn	To identify common and unique aspects of early social communication by investigating descriptive patterns, differences, and the relationship with ASD risk and early social communication complexity across, within, and between IL-FXS, IL-Sibs, and LL controls	IL-FXS: Confirmed diagnosis of FXS by genetic report IL-Sibs: Documentation of ASD diagnosis for an older full sibling LL: ns	ns	No ASD outcome assessment was conducted; study was cross-sectional and assessed study participants between 7.5 and 14.5 months. ASD symptomology was measured using the AOSI
2017 Bedford δ	To test whether infant neurocognitive markers (indexing eye-gaze processing and attention control) and 7/14-month AOSI scores can distinguish between IL-Sib 7-year ASD diagnostic status	IL-Sibs: Have an older full/half sibling with a community clinical diagnosis of ASD confirmed using the DAWBA and SCQ by expert clinicians LL: Have an older full/half sibling that does not meet criteria for ASD on the SCQ (does not meet instrument cut-off of ≥ 15)	ns	36-month ASD assessment: ns 7-year ASD assessment: Non-blinded diagnostic assessment using DSM-5 criteria for ASD based on all previous study information (ADOS-2, ADI-R, WASI-II, VABS-II) conducted by four experienced researchers
2017 McDonald β	To investigate whether (1) delays in social communication as measured by the AOSI may be observed within the first year of life for IL-TSC infants, and (2) if such delays are related to later ASD diagnostic status	For all participants: Availability of AOSI and cognitive functioning data at 9 and/or 12 months, clinical outcome data at 18, 24, and/or 36 months IL-TSC: Be diagnosed or present with TSC (based on clinical presentation). LL: ns	IL-TSC: ns LL: prematurity (< 37 weeks gestational age), birth trauma, developmental concerns, or close family history of ASD or intellectual disability	Clinical best estimate diagnosis based on ADOS scores. ASD outcome assessment was made at either 18-, 24-, or 36-months depending on availability of ADOS data (if a child had multiple clinical ASD outcome visits, the most recent ADOS score was used in ASD determination)
2018 Bussu δ	To (1) investigate longitudinal differences from 8–36 months between IL-Sibs with different developmental outcomes (typical, atypical, ASD) and LL controls, and (2) predict ASD or atypical development at 36-months an individual level for IL-Sibs using supervised machine learning classifier analysis based on 8- and 14-month study data	IL-Sibs: Have an older biological sibling with ASD LL: Have an older full sibling with typical development	All participants: Lack of 36-month ADOS and/or 36-month clinical outcome evaluation	36-month clinical consensus best estimate diagnosis considering 24 (ADOS, MSEL, VABS) and 36-month study data (ADOS, ADI-R, MSEL, VABS) using ICD-10 or DSM-5 criteria (dependent on study phase). Categorization of ASD using ICD-10 (atypical autism, PDD-unspecified, PDD-other) and DSM-5 criteria was considered similar following a review of ASD diagnoses by the clinical research lead
2018 Sacrey α	To examine the agreement between parent and clinician ratings (on the APSI and AOSI respectively) regarding early symptoms of ASD in a sample of IL-Sibs	IL-Sibs: Have an older sibling formally diagnosed with ASD that was confirmed by clinical assessment or a review of diagnostic records using DSM-IV-TR criteria, have undergone a 36-month diagnostic assessment for ASD, have APSI and AOSI outcome data at 12 and/or 18-months	All participants: Born prior to 36 weeks gestation, birth weight < 2500 g, identifiable neurological or genetic conditions, or severe sensory or motor impairments	36-month blind, independent best judgement diagnostic assessment using DSM-IV-TR criteria that considered ADOS, ADI-R, MSEL, and VABS data. Diagnoses were assigned by an expert clinician (developmental pediatrician, child psychiatrist, or clinical psychologist) with 10 + years of diagnostic experience
2019 Bedford δ	To test the hypothesis that (1) infant regulatory function is negatively associated with traits of ASD, ADHD, but not callous unemotional traits, and (2) that regulatory function moderates the association between known infant markers (activity level for ADHD, early autism-like behaviours measured on the AOSI) with later traits of ADHD and ASD	IL-Sibs: Have an older full/half sibling with a community clinical diagnosis of ASD, confirmed using the DAWBA and SCQ by expert clinicians LL: Have an older full/half sibling that does not meet criteria for ASD on the SCQ (does not meet instrument cut-off of ≥ 15)	ns	7-year ASD assessment: Diagnostic assessment using DSM-5 criteria was based on ADOS-2, ADI-R, SCQ, VABS-II, and WASI-II study data. Diagnoses were assigned by four experienced researchers following a review on ASD symptomatology
2020 Hahn γ	To describe ASD-associated behaviours in IL-DS infants 7–18 months old relative to LL controls 9–14 months old	IL-DS: Recruited from three pilot studies examining infant phenotype in neurogenetic syndromes who recruited participants by flyers shared with parent groups, DS clinics, and/or other research studies LL: Recruited from another study on the emergence of ASD in FXS. LL controls were matched to IL-DS based on sex at an individual level, and age at a group level	IL-DS: ns LL: No ASD or other developmental disability (how this determination was made was not specified)	No ASD outcome assessment was conducted; study was a case–control cross-sectional study that assessed IL-DS 7–18 months old and LL controls 9–14 months old. ASD symptomology was instead measured using the AOSI
2020 Zwaigenbaum α	To characterize behavioural signs of ASD in IL younger siblings of children on the autism spectrum and examine classification features of the AOSI	IL-Sibs: Have an older sibling formally diagnosed with ASD confirmed by clinical assessment or a review of records using DSM-IV-TR criteria LL: No 1^st^ or 2^nd^ degree relative with ASD	All participants: Born < 36 weeks gestation, birth weight < 2500 g, identifiable neurological or genetic conditions, severe sensory or motor impairments	36-month blind, independent diagnostic assessment using DSM-IV-TR criteria was based on ADOS, ADI-R data and a review of other developmental assessments (MSEL, VABS)
2021 Zwaigenbaum α	To (1) identify distinct trajectories of ASD symptoms indexed by AOSI data from 6–18 months assessments, (2) examine the relationship between AOSI-informed trajectory group membership and 3-year clinical outcomes, and (3) to compare clinical features among IL-Sibs diagnosed with ASD across each trajectory with respect to sex ratio, language, cognitive and adaptive skills, and ASD symptom severity	IL-Sibs: Have an older sibling diagnosed with ASD confirmed by clinical assessment or a review of diagnostic records using DSM-IV-TR criteria LL: No 1^st^ or 2^nd^ degree relative with ASD	All participants: Born < 36 weeks gestation, birth weight < 2500 g, identifiable neurological or genetic conditions, severe sensory or motor impairments	36-month independent, clinical best estimate diagnostic assessment using DSM-IV-TR criteria was based on all available study data (ADOS, ADI-R, MSEL, VABS) were conducted by an expert clinician blinded to prior study assessments. IL-Sibs and LL controls not meeting diagnostic criteria for ASD were further stratified into a ‘delays or differences’ category if they scored > 1.5 SD below the mean on ≥ 1 MSEL subscales and/or if they scored > 3 on the ADOS calibrated severity score

ADI-R = Autism Diagnostic Interview-Revised, ADHD = Attention Deficit Hyperactivity Disorder, ADOS = Autism Diagnostic Observation Schedule, APSI = Autism Parent Screen for Infants, ASD = Autism Spectrum Disorders, CCS = communication complexity scale, DAWBA = Developmental and Wellbeing Assessment, DS = Down Syndrome, DSM = Diagnostic and Statistical Manual of Mental Disorders, FXS = Fragile X Syndrome, ICD-10 = International Classification of Diseases, 10^th^ revision, IL = Infants at increased likelihood for ASD, IL-FXS = Infants diagnosed with Fragile X Syndrome, IL-TSC = Infants diagnosed with Tuberous Sclerosis Complex, IL-DS = Infants diagnosed with Down Syndrome, IRB = Institutional Review Board, ns = not specified, MRI = magnetic resonance imaging, PDDNOS = Pervasive Developmental Disorder Not Otherwise Specified, Proband = an IL-Sibs older sibling who is either diagnosed with or meets criteria for ASD, SCQ = Social Communication Questionnaire, SD = Standard deviation, TSC = tuberous sclerosis complex, VABS = Vineland Adaptive Behaviour Scale, WASI-II = Wechsler Abbreviated Scale of Intelligence-Second Edition

α = Conducted on Canadian Infant Sibling Study [CISS] participants

β = Conducted using some of the same study participants

γ = Conducted using some of the same study participants

δ = Conducted on British Autism Study in Infant Siblings [BASIS] participants

#### ASD outcome assessment

The assessment of ASD varied across the 17 included studies. Of the five studies (Capal et al., 2017, Zwaigenbaum et al., 2005, Estes et al., 2015, Roberts et al., 2016, Gliga et al. 2015) using 24-month ADOS scores as an outcome measure of ASD symptoms, only one (Estes et al., 2015) conducted 24-month clinical best estimate diagnostic assessments using 24-month ADOS, ADI-R scores, and DSM-IV-TR criteria (ASD or pervasive developmental disorder [PDD] not otherwise specified). Eight studies (Bedford et al., 2016; Bussu et al., 2018; Bussu et al., 2018; Gammer et al., 2015; Jeste et al., 2014; McDonald et al., 2017; Sacrey et al., 2018; Zwaigenbaum et al., 2021a, 2021b) conducted 36-month ASD diagnostic assessments, though their assessment modalities varied. Bussu et al., 2018, Zwaigenbaum et al. 2020, Zwaigenbaum et al., 2021a, 2021b, and Sacrey et al., 2018 conducted independent or clinical consensus best estimate ASD diagnostic assessments based on ADOS, ADI-R, and cognitive, language, or developmental scales (MSEL, VABS) using ICD-10 (atypical autism, PDD-unspecified, PDD-other; Bussu et al., 2018) or DSM diagnostic criteria (Zwaigenbaum et al. 2020, Zwaigenbaum et al., 2021a, 2021b, Sacrey et al., 2018). Gammer et al. (2015) conducted assessments based on ADOS and ADI-R data using ICD-10 diagnostic criteria (childhood autism, PDD), Bedford et al. (2016) based on ADOS and Social Communication Questionnaire (SCQ) data using ICD-10 criteria for autism (childhood autism, PDD), and McDonald et al. (2017) made clinical best estimate diagnoses based on ADOS data with no mention of using DSM or ICD-10 criterion. Jeste et al. (2014) assigned ASD diagnoses based on convergence of ADOS scores (taken at 18-, 24-, and 36-month assessments) and clinical judgement with no mention of ICD-10 or DSM criterion. Two studies, Bedford et al. (2017) and Bedford et al. (2019), focused on ASD outcomes in early-to-mid childhood and conducted 7-year ASD diagnostic assessments using ADOS, ADI-R, and cognitive, language, or developmental scales (VABS-II, WASI-II). Finally, the remaining two studies (Hahn et al., 2017; Hahn et al., 2017) were cross-sectional in nature and did not assess for ASD outcomes (ASD diagnoses were not applicable based on their study objectives).

#### Age at AOSI Administration

Three studies administered the AOSI at 12- or 14-month time points (Bedford et al., 2016; Capal et al., 2017; Roberts et al., 2016). Two studies, Hahn et al. (2020) and Hahn et al. (2017), administered the AOSI over a wide range of ages (7–18 months) instead of at a specified time point. The remaining 12 studies administered the AOSI over multiple time points between 6 and 18 months.

#### Calculating AOSI Total Scores or AOSI Risk Markers

The AOSI can be scored using two different metrics: the AOSI Total Score constituting a summed score of items 1 to 18 on the scale, and AOSI Risk Markers constituting a tally of AOSI items 1 to 18 that score at least a 1 or higher (Bryson et al., 2008; Zwaigenbaum et al., 2005). It is important to note that these metrics are *not* the same thing. While 15 of 17 studies in this review calculate AOSI Total Scores for IL or LL study participants (barring Hahn et al., 2020 and Zwaigenbaum et al., 2005), only 2 of 17 studies report calculated AOSI Risk Marker scores (Hahn et al., 2020; Roberts et al., 2016).

#### AOSI Metrics Used in Sensitivity and Specificity Estimates

Overall, only six studies report whether or not they employed or calculated AOSI Total Score (Capal et al., 2017, Zwaigenbaum et al. 2020, Hahn et al., 2017) or AOSI Risk Marker cut points (Hahn et al., 2020; Roberts et al., 2016; Zwaigenbaum et al., 2005). Of these six studies, only four (Capal et al., 2017, Zwaigenbaum et al., 2005, Zwaigenbaum et al. 2020, Roberts et al., 2016) directly report their corresponding psychometric estimates (sensitivity/specificity) or the data needed to calculate them. Two studies (Roberts et al., 2016; Zwaigenbaum et al., 2005) used AOSI Risk Markers for their psychometric estimates, and two (Capal et al., 2017, Zwaigenbaum et al. 2020) used AOSI Total Scores.

How AOSI Total Scores or AOSI Risk Markers have been used in these four studies varied as no consistent cut point for either metric was employed. Two studies, Zwaigenbaum et al. (2005) and Roberts et al. (2016), used a cut-point of ≥ 7 or > 7 AOSI Risk Markers respectively to predict 24-month ASD classification whereas Capal et al. (2017) and Zwaigenbaum et al. (2020) computed multiple AOSI Total Score cut points to predict 24-month or 36-month ASD classification or diagnosis respectively. That is, Capal et al. (2017) provided a range of possible Total Score cut points based on 12-month assessment data while Zwaigenbaum et al. (2020) computed a range of possible Total Score cut points for each time point they administered the AOSI (6, 9, 12, 15, and 18 months).

Though not reporting AOSI cut points and their corresponding psychometric estimates, Zwaigenbaum et al., (2021a, 2021b) imported AOSI Total Score data from participants assessed at 6, 9, 12, 15, and 18 months into STATA to generate semi-parametric group-based trajectory models that reflect sub-populations of participants. After selecting for a 3-group quadratic model, Zwaigenbaum et al., (2021a, 2021b) compared participant membership in these groups (Group 1 = ‘Low and stable,’ Group 2 = ‘Intermediate and stable,’ and Group 3 = ‘Inclining’) in their trajectory model against later 36-month ASD diagnostic outcomes (IL siblings diagnosed with ASD, IL siblings not diagnosed with ASD, LL controls). While not reporting AOSI cut points and their corresponding psychometric estimates, the sensitivity and specificity of these trajectory models relative to 36-month ASD outcomes was documented. Table 3 provides more details.

**Table 3:** AOSI Analyses and Psychometric Estimates

Article	How was the AOSI used/applied?	Total Score or Risk Marker?	IL Group	Timepoint	Cut Point	Sensitivity	Specificity
2005 Zwaigenbaum *	AOSI data taken from 6 and 12-month assessments was compared against IL/LL study participants based on 24-month ADOS classification using One-way ANOVA analysis with follow-up multiple comparisons. 12-month AOSI scores were used to predict 24-month ADOS classification	Risk Markers	IL-Sibs	12 months	≥ 7	0.84	0.98
2016 Roberts	12-month AOSI scores were explored across IL/LL groups using one-way Kruskal Wallis analysis with Dunn post hoc pairwise comparisons. Fischer’s exact test was used to investigate (1) group differences in the proportion of IL/LL infants who flagged positive on the AOSI relative to those who did not, and (2) item-level group differences. 12-month AOSI scores were used to predict 24-month ADOS classification	Risk Markers	IL-FXS	12 months	> 7	0.57	1.00
			IL-Sibs	12 months	> 7	1.00	0.57
2017 Capal	12-month AOSI scores were used as a predictor variable in logistical regression models against 24-month ADOS-2 and ADI-R outcome data (IL-TSC participants classified with/without ASD). 12-month AOSI Total Score cut points were examined with respect to later 24-month ADOS classification	Total Score	IL-TSC	12 months	8	0.67	0.70
					9	0.67	0.73
					10	0.58	0.77
					11	0.51	0.82
					12	0.48	0.82
					13	0.39	0.89
					14	0.36	0.91
2020 Zwaigenbaum *	AOSI data taken from 6, 9, 12, 15, and 18-month assessments were compared using linear mixed modelling. ROC curve analysis assessed longitudinal associations between AOSI Total Score data at each timepoint with later 36-month clinical outcomes. Optimal Total Score cut points were calculated using Youden indices. AOSI scoring data was compared across IL-Sibs with/without ASD, with Fischer’s exact test calculated to compare the percentage of IL-Sibs correctly identified at 24- and 36-month assessments	Total Score	IL-Sibs	6 months	7	0.57	0.51
				9 months	8	0.60	0.53
				12 months	7	0.52	0.74
				15 months	10	0.41	0.90
				18 months	6	0.73	0.65
2021 Zwaigenbaum *	Trajectory modeling based AOSI Total Scores data was derived using Stata group-based modelling approach on data taken at 6, 9, 12, 15, and 18-month assessments. The relationship between AOSI trajectory group membership in the finalized trajectory model and 36-month clinical outcomes was examined to assess accuracy of group membership relative to ASD diagnosis. Clinical features of participants diagnosed with ASD at 36 months were compared by trajectory group using one-way ANOVAs	Longitudinal Total Score data from 6–18 months	IL-Sibs	n/a	Group 1	0.28	0.94
				n/a	Group 2	0.68	0.59

ADI-R = Autism Diagnostic Interview-Revised, ADOS = Autism Diagnostic Observation Schedule, ANOVA = Analysis of Variance, AOSI = Autism Observation Scale for Infants, IL = infants at increased likelihood for ASD diagnosis, IL-FXS = IL infants with Fragile X Syndrome, IL-TSC = IL infants with Tuberous Sclerosis Complex, IL-Sibs = IL infant siblings, LL = infants at low likelihood for ASD diagnosis, ROC = receiver operating characteristic

^*^ = Conducted on Canadian Infant Sibling Study [CISS] participants

For additional methodological considerations including article study design, AOSI reliability data (inter-rater, item-level agreement between coders, etc.), how infant sibling studies defined the older sibling (proband) as having ASD, and what inclusion/exclusion criteria were employed, see Supplementary File 3.

### Main Findings

#### AOSI Sensitivity and Specificity Estimates for Infant Siblings

The cut points and AOSI metrics used varied across studies which makes it difficult to compare sensitivity, as described in Table 3. Of the four studies which assessed infant siblings, two studies using AOSI Risk Marker cut points of ≥ 7 or > 7 (Roberts et al., 2016; Zwaigenbaum et al., 2005) had sensitivity estimates of 0.84 and 1.00 respectively. Zwaigenbaum et al. (2020), who assessed different AOSI Total Score cut points across a range of time points had sensitivity values ranging between 0.41 and 0.73. For Zwaigenbaum et al., (2021a, 2021b) who used trajectory-based grouping based on AOSI Total Scores, sensitivity estimates for the inclining trajectory and inclining + intermediate trajectory groups were 0.28 and 0.68 respectively.

Though specificity estimates were largely higher than sensitivity estimates for infant siblings, variation was still noted. Two studies that used AOSI Risk Marker cut points of ≥ 7 or > 7 (Roberts et al., 2016; Zwaigenbaum et al., 2005) reported specificity estimates of 0.98 and 0.57 respectively. Zwaigenbaum et al. (2020), who assessed different AOSI Total Score cut points across a range of time points reported specificity estimates ranging between 0.51 and 0.90. For Zwaigenbaum et al., (2021a, 2021b) who used trajectory-based grouping based on AOSI Total Scores, specificity estimates for the inclining trajectory and inclining + intermediate trajectory groups were 0.94 and 0.59 respectively.

#### AOSI Sensitivity and Specificity Estimates for FXS and TSC Infants

In addition to there being fewer psychometric estimates available for FXS and TSC infants, cut points and metric used varied relative to infant siblings as described in Table 3. Using the AOSI Risk Marker cut point of > 7, Roberts et al. (2016)’s data led to a single calculated sensitivity estimate of 0.57 for FXS infants. For Capal et al. (2017) who report a range of 12-month AOSI Total Score cut points in TSC infants, sensitivity estimates ranged between 0.36 and 0.67.

Specificity estimates for infants with FXS and TSC resembled those for infant siblings. Using the AOSI Risk Marker cut point of > 7, Roberts et al. (2016)’s data led to a single calculated specificity estimate of 1.00 for FXS infants. For Capal et al. (2017), specificity estimates for a variety of 12-month AOSI Total Score cut points ranged between 0.70 and 0.91.

#### AOSI Total Score Comparison

**Fig. 2:**
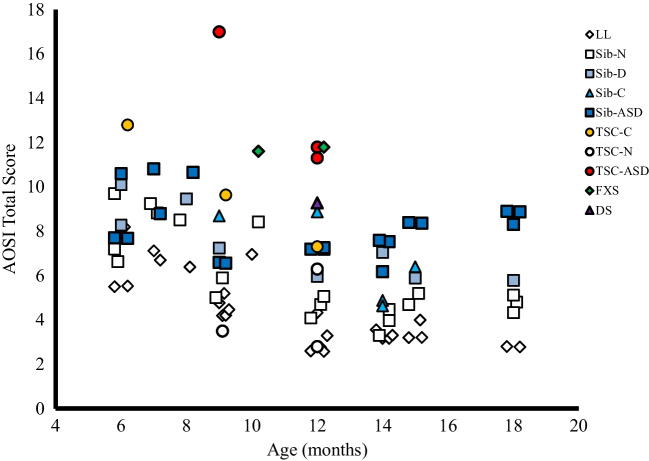
Scatterplot of age (in months) by AOSI Total Score. Note that while different IL-ASD groups are denoted by the filled symbols, LL and IL non-ASD groups are denoted by the open symbols. ASD = autism spectrum disorders, DS = infants with Down syndrome, FXS = infants with Fragile X syndrome, LL = low likelihood control infants, Sibs-ASD = infant siblings diagnosed with ASD, Sibs-C = combined infant sibling group (ASD not separated out), Sibs-D = infant siblings who are developmentally delayed, Sibs-N = infant siblings not diagnosed with ASD, TSC = Tuberous sclerosis complex, TSC-ASD = infants with TSC diagnosed with ASD, TSC-C = combined TSC group (ASD not separated out), TSC-N = infants with TSC not diagnosed with ASD

As shown in Fig. 2 (scatterplot), a consistent pattern of AOSI Total Scores emerges at 12 months of age, with IL-ASD groups (TSC, FXS, DS, and Infant Siblings with ASD) consistently showing higher scores compared to LL and IL non-ASD comparison groups.

## Meta-Analyses

### LL Controls and IL-ASD

#### Between 6 and 10 months

**Fig. 3:**
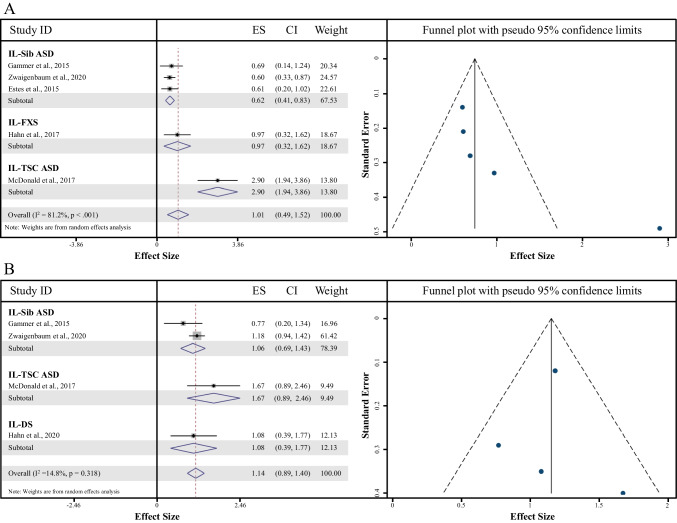
**a, b |** Meta-Analysis comparing LL Controls to IL-ASD Samples (left) with the Trim and Fill Plot (right). A = for ages 6–10 months, B = for ages 12–14 months

A total of five comparisons of AOSI Total Scores were included in this meta-analysis. There was a significant effect of AOSI Total Score, suggesting that the IL-ASD group had higher AOSI Total Scores compared to the LL control group (Cohen’s *d* = 1.01, 95% CI = 0.49—1.52, *z* = 3.82, *p* < 0.001, Fig. 3a). High heterogeneity was seen among the included studies (*I*^*2*^ heterogeneity statistic = 81.2%); thus, a random effects model was adopted to pool the relevant data and explore subgrouping analyses to determine any differential effects of the IL-ASD subgroup on AOSI Total Score. As shown in Fig. 3a, all three IL-ASD groups (Sib-ASD, FXS, and TSC-ASD) produced significant effects (all *p’*s < 0.01), resulting in higher AOSI Total Scores compared to LL controls. Funnel plot analyses on Cohen’s *d* for AOSI Total Score demonstrated symmetry, but we still assessed for the presence of bias (Fig. 3a). Trimming the set of data systematically removes each ‘outlier’ one at a time and recalculates the resulting Cohen’s *d*. The resultant value was changed following the trim and fill analyses, suggesting 2 missing studies. Evaluation of the Egger test provided little evidence of small study effects impacting Cohen’s *d* (bias coefficient = 5.43, standard error = 2.36; *t* = 2.30, *p* = 0.15).

#### Between 12 and 14 months

A total of four comparisons of AOSI Total Scores were included in this meta-analysis. There was a significant effect of AOSI Total Score, suggesting that the IL-ASD group (Sib-ASD, DS, and TSC-ASD) had higher AOSI Total Scores compared to the LL control group (Cohen’s *d* = 1.15, 95% CI = 0.90—1.40, *z* = 8.96, *p* < 0.001, Fig. 3b). Though low heterogeneity was seen among the included studies (*I*^*2*^ heterogeneity statistic = 14.8%); we still adopted a random effects model to pool relevant data and explore subgrouping analyses to determine any differential effects of the IL-ASD subgroup on AOSI Total Score. As shown in Fig. 3b, all three IL-ASD groups (Sib-ASD, DS, and TSC-ASD) produced significant effects (all *p’*s < 0.03), resulting in higher AOSI Total Scores compared to LL controls. Though funnel plot analyses on Cohen’s *d* for AOSI Total Score demonstrated symmetry, we still assessed for the presence of bias (Fig. 3b). The Cohen’s *d* value was unchanged following the trim and fill analyses, suggesting no bias. Evaluation of the Egger test provided little evidence of small study effects impacting Cohen’s *d* (bias coefficient = -0.01, standard error = 1.37; *t* = 0.00, *p* = 0.99).

### IL Non-ASD Combined Controls and IL-ASD

#### Between 6 and 10 months

**Fig. 4:**
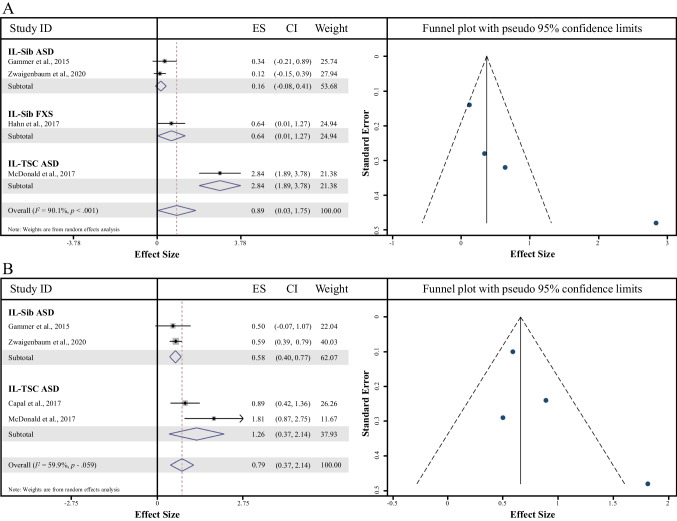
**a, b** | Meta-Analysis comparing IL non-ASD Controls to IL-ASD Samples (left) with the Trim and Fill Plot (right). A = for ages 6–10 months, B = for ages 12–14 months

A total of four comparisons of AOSI Total Scores were included in this meta-analysis. There was a significant effect of AOSI Total Score, suggesting that the IL-ASD group had higher AOSI Total Scores compared to the IL control group (Cohen’s *d* = 0.89, 95% CI = 0.03—1.75, *z* = 2.02, *p* = 0.004, Fig. 4a). High heterogeneity was seen among the included studies (*I*^*2*^ heterogeneity statistic = 90.1%); thus, a random effects model was adopted to pool the relevant data and explore subgrouping analyses to determine any differential effects of the IL-ASD subgroup on AOSI Total Score. As shown in Fig. 4a, two of the three IL-ASD groups produced significant effects resulting in higher AOSI Total Scores compared to IL controls for FXS (*p* = 0.05) and TSC-ASD (*p* < 0.001). Funnel plot analyses on Cohen’s *d* for AOSI Total Score demonstrated symmetry, but we assessed for the presence of bias regardless (Fig. 4a). The Cohen’s *d* value was changed following trim and fill analyses, suggesting 2 missing studies. Evaluation of the Egger test provided little evidence of small study effects impacting Cohen’s *d* (bias coefficient = 5.43, standard error = 2.36; *t* = 2.30, *p* = 0.15).

#### Between 12 and 14 months

A total of four comparisons of AOSI Total Scores were included in the meta-analysis. There was a significant effect of AOSI Total Score, suggesting that the IL-ASD group had higher AOSI Total Scores compared to the IL control group (Cohen’s *d* = 0.79, 95% CI = 0.42—1.17, *z* = 4.15, *p* < 0.001, Fig. 4b). Moderate heterogeneity was seen among the included studies (*I*^*2*^ heterogeneity statistic = 59.9%); thus, a random effects model was adopted to pool relevant data and explore subgrouping analyses to determine any differential effects of the IL-ASD subgroup on AOSI Total Score. As shown in Fig. 4b, both IL-ASD groups (Sib-ASD and TSC-ASD) produced significant effects (all *p’*s < 0.01), resulting in higher AOSI Total Scores compared to IL controls. Funnel plot analyses on Cohen’s *d* for AOSI Total Score demonstrated symmetry, but we assessed for the presence of bias regardless (Fig. 4b). The Cohen’s *d* value was unchanged following the trim analyses, but the fill analysis suggested there was 1 missing study. Evaluation of the Egger test provided little evidence of small study effects impacting Cohen’s *d* (bias coefficient = 1.91, standard error = 1.26; *t* = 1.52, *p* = 0.27).

### IL-DD/IL-Typical and IL-ASD

Meta-analyses were also performed on studies that broke the IL-N participants who were not diagnosed with ASD into groups which met criteria for developmental delay (IL-DD) and those who showed typical development (IL-Typical). These data are presented in Supplementary File 3.

### Checklist of Bias and Quality of Study Methodology

Table 4 provides a visual overview of the methodological strengths and weaknesses of the 17 studies included in this review. In total, KR and LS had 97.41% agreement when scoring the composite checklist with disagreements resolved via consensus discussion. Overall, there was no consistent approach with respect to classification or diagnosis of ASD (both for age and measures used), inclusion or exclusion criteria for participants, choice of comparison groups (or lack thereof), whether AOSI item-level, Risk Marker, or Total Score data are reported, and participant demographics (age, SES, ethnicity, parental age, etc.). A consideration of each of these factors is important when making methodological decisions.

**Table 4:** Bias and Quality Checklist for Study Methodology

	Zwaigenbaum 2005	Jeste 2014	Estes 2015	Gammer 2015	Gliga 2015	Bedford 2016	Roberts 2016	Capal 2017	Hahn 2017	Bedford 2017	McDonald 2017	Bussu 2018	Sacrey 2018	Bedford 2019	Hahn 2020	Zwaigenbaum 2020	Zwaigenbaum 2021
Objective / purpose																	
Question	✓	✓	✓	✓	✓	✓	✓	✓	✓	✓	✓	✓	✓	-	✓	✓	✓
Hypothesis	-	-	-	-	✓	-	-	-	-	-	✓	✓	-	✓	-	-	-
Study Design																	
Cross-sectional	-	-	-	-		-	-	-	✓	-	-	-	-	-	✓	-	-
Longitudinal	✓	✓	✓	✓	✓	✓	✓	✓	-	✓	✓	✓	✓	✓	-	✓	✓
Exclusion Criteria																	
Birth weight	-	-	✓	-	✓	-	-	-	-	-	-	-	✓	-	-	✓	✓
Term birth	-	✓	✓	-	✓^Θ^	-	✓	✓	-	✓^Θ^	✓	-	✓	-	-	✓	✓
Genetic causes	-	-	-	✓	-	-	✓	-	-	-	-	-	✓	-	-	✓	✓
Other conditions	-	✓	✓	✓	-	-	✓	✓	-	-	✓	-	✓	-	-	✓	✓
Recruitment																	
Same cohort	✓	✓	-	✓	✓*	✓*	✓	✓	✓	-	-	-	✓	✓	-	✓	✓
Sample calculation	-	-	-	-	-	-	-	-	-	-	-	-	-	-	-	-	-
Control group																	
LL controls	✓	✓	✓	✓	✓	✓	✓	-	✓	✓	✓	✓	-	✓	✓	✓	✓
IL controls	✓	✓	✓	✓	-	-	✓	✓	✓	✓	✓	✓	✓	-	-	✓	✓
Experimental group																	
Infant siblings	✓	-	✓	✓	✓	✓	✓	-	-	✓	-	✓	✓	✓	-	✓	✓
Infants with FXS	-	-	-	-	-	-	✓	-	✓	-	-	-	-	-	-	-	-
Infants with TSC	-	✓	-	-	-	-	-	✓	-	-	✓	-	-	-	-	-	-
Infants with DS	-	-	-	-	-	-	-	-	-	-	-	-	-	-	✓	-	-
Demographics																	
Sex	-	-	✓	✓	✓	✓	✓	✓	✓	✓	✓	✓	✓	✓	✓	✓	✓
SES	-	-	-	✓	-	-	-	-	-	✓^#^	-	-	✓^#^	-	-	✓	✓
Ethnicity	-	-	✓	-	-	-	-	-	✓	-	✓	-	-	-	✓	✓	✓
Parental age	-	-	-	-	-	-	-	-	-	-	-	-	✓^#^	-	-	-	-
Outcome Assessment																	
2 years	✓	-	✓	-	✓	-	✓	✓	-	✓	✓	✓		✓^Δ^	-	-	-
3 years	-	✓	-	✓	-	✓	-	-	-	✓	✓	✓	✓	✓^Δ^	-	✓	✓
7 years	-	-	-	-	-	-	-	-	-	✓	-	-	-	✓	-	-	-
Gold-standard?	-	-	-	✓	-	-	-	✓	-	✓	✓	✓	✓	✓	-	✓	✓
Blinded?	-	-	✓^α^	-	-	-	-	✓^α^	-	✓	-	✓	✓	-	-	✓	✓
Diagnostic Criteria																	
DSM	-	-	✓	-	-	-	-	-	-	✓	-	✓	✓	✓	-	✓	✓
ICD	-	-	-	✓	-	✓	-	-	-	-	-	✓	-	-	-	-	-
Age of AOSI Administration																	
< 12 months	-	✓	✓	✓	✓		-	-	-	✓	✓	✓	-	✓	-	✓	✓
≥ 12 months	✓	✓	✓	✓	✓	✓	✓	✓	-	✓	✓	✓	✓	✓	-	✓	✓
Range < 12 >	-	-	-	-	-	-	-	-	✓	-	-	-	-	-	✓	-	-
AOSI Administrations																	
One	-	-	-	-	-	✓	✓	✓	✓	-	-	-	-	✓	✓	-	-
Two +	✓	✓	✓	✓	✓	-	-	-	-	✓	✓	✓	✓	-	-	✓	✓
Statistical analyses																	
Covariates	-	-	✓	✓	-	✓	✓	✓	✓	-	✓	✓	✓	✓	-	-	-
Post-hoc	✓	-	✓	✓	-	-	✓	-	✓	-	✓	✓	-	-	-	✓	✓
Included in meta-analysis?	-		✓	✓	-	-		✓	✓	-	✓	✓	-	-	✓	✓	✓
AOSI content used																	
Item-level data	-	-	-	✓	-	-	✓	✓	-	-	✓	-	✓	-	-	✓	-
Total Score data	✓	✓	✓	✓	✓	✓	✓	✓	✓	✓	✓	✓	✓	✓	✓	✓	✓
Psychometric properties																	
Sensitivity	✓	-	-	-	-	-	✓	✓	-	-	-	-	-	-	-	✓	✓
Specificity	✓	-	-	-	-	-	✓	✓	-	-	-	-	-	-	-	✓	✓
Cut-off score	✓	-	-	-	-	-	✓	✓	-	-	-	-	✓	-	-	✓	✓
IRR assessment?	✓	-	-	-	-	✓	✓	-	✓	✓	-	-	-	✓	-	-	-

AOSI = Autism Observation Scale for Infants, DS = Down Syndrome, DSM = Diagnostic and Statistical Manual of Mental Disorders, FXS = Fragile X Syndrome, ICD = International Classification of Diseases, IRR = inter-rater reliability, SES = socioeconomic status, TSC = Tuberous Sclerosis Complex, ^α^ = Not all clinicians responsible for assigning a diagnosis were blinded group status. Note: checkmarks for each item are not weighted and instead simply denote the presence or absence of the item

^*^ = Control sample came from volunteer databases, but it is not clear whether they were recruited from the same cohort as IL participants

Θ = Term birth was described only for control participants

^#^ = Family income was calculated and used in statistical calculations but not described or reported (i.e., no direct reporting of SES for IL vs LL groups)

Δ = While participants were described as being at these ages, the assessment procedure was not detailed or described outside of being mentioned

## Discussion

This systematic review and meta-analysis focused on previous studies assessing classification properties and group differences on the AOSI across different IL infant populations. Four IL populations were identified in this review: infants with FXS, TSC, DS, and infant siblings of children on the autism spectrum. The review had three main findings. First, although five studies reported individual classification properties, sensitivity and specificity estimates were not comparable due to the different metrics, methodologies, and cut point scores used. Second, stable group differences emerged between LL and IL non-ASD control groups and IL-ASD groups by 12 months of age. Third, meta-analyses identified a large effect size for comparisons between LL control and IL-ASD samples, and a moderate effect size for comparisons of IL non-ASD and IL samples with signs or diagnoses of ASD. Gaining a better understanding of how the AOSI performs across different populations of infants who are at increased likelihood for ASD has important implications for our understanding and characterization of the emergence of ASD during early childhood.

### Methodological Concerns Regarding Classifying and Diagnosing ASD in IL Samples

ASD outcomes were assigned based on either 24-month ADOS classification or 36-month blinded diagnostic assessments. When assessing for ASD, the age of the child and the comprehensiveness of the assessment are important. Infant behaviour can be affected by situational factors, such as their state of alertness (Jones et al., 2014), time of day, and/or biological state (e.g., hunger or sleepiness; McNally et al., 2016). Gold-standard ASD diagnostic assessments (defined as use of validated observational and interview measures such as the ADOS and ADI-R in conjunction with expert clinical judgement; Kaufman, 2022) utilize a broad scope of clinical information before assigning a diagnosis. Use of a single observational measure to determine ASD outcome is therefore a poor proxy and likely suffers from decreased sensitivity, specificity, and diagnostic stability (Jones et al., 2014). Furthermore in IL infant siblings, although diagnostic stability of early ASD diagnosis at 18- and 24-months is high at 93% and 82% respectively, early classification suffers from low sensitivity (Ozonoff et al., 2015). At 18- and 24-month assessments, 63% and 41% of children who are later diagnosed with ASD at 36-months are missed (Ozonoff et al., 2015). Since 24-month clinical best estimate ASD diagnosis can miss such a substantial percentage of children later diagnosed at 36-months, 24-month classification of ASD based on ADOS scores alone are likely even less accurate.

### Validation of the AOSI in Different IL Samples

When extending the use of an established scale to a new context, caution must be practiced; it cannot be assumed that a scale validated in one population can be equally applied in a different population without initial validation (Streiner et al. 2014). Each time a scale is used in a new context, it is necessary to establish psychometric properties and validity of the inferences drawn from them (Streiner et al., 2015). In addition, in pursuit of optimal reliability and validity, scales often need to be revised – changes may be subtle or substantial (Streiner et al., 2015). For example, FXS infants with ASD have significantly higher motor impairments relative to infant siblings with ASD (Roberts et al., 2016). Whether such variance in item-level scoring is present across the different IL populations is not clear. Possible alterations to the AOSI may be warranted to capture population differences that may be indicative of later ASD diagnoses. We suggest that item-level data should be reported to assist this effort.

Sensitivity, the ability of a test to correctly identify an individual as having a particular condition, and specificity, the capability of a test to correctly identify individuals as not having that condition, are inversely proportional (Parikh et al., 2008). The AOSI cut point should optimize both sensitivity and specificity (Akobeng, 2007). Although the best tests are both highly sensitive and specific, this is not always feasible in practice (Akobeng, 2007) as trade-offs may exist between valuing high sensitivity over specificity (or vice versa, Trevethan, 2017). In situations where it is vital that a diagnosis is not missed (e.g., diseases with high mortality), high sensitivity is sought. In contrast, if the consequences of false positives are serious (e.g., psychological implications of a false HIV diagnosis), high specificity is sought (Akobeng, 2007).

AOSI sensitivity and specificity estimates for infant siblings varied across the papers reviewed here. Although Zwaigenbaum et al. (2005) and Roberts et al. (2016) used a similar cut point (≥ 7 and > 7 AOSI Risk Markers respectively), their estimates of specificity differed. This likely stemmed from study differences in inclusion/exclusion criteria, participant demographics, and use of 24-month classification assessment (which may be less sensitive to children with milder ASD presentation). The issue of psychometric properties is further muddied by the AOSI metric used. Rather than AOSI Risk Markers, sensitivity and specificity estimates from Zwaigenbaum et al. (2020) were calculated using the AOSI Total Score, which may account for differences in sensitivity and specificity. The original Zwaigenbaum et al. (2005) article introducing the AOSI published preliminary psychometric estimates based on a cut point of ≥ *7 AOSI Risk Markers*, not the AOSI Total Score. The two metrics are not comparable. AOSI Risk Markers denote the total number of AOSI items that scored ‘1’ or higher and range from 0–16 (Zwaigenbaum et al., 2005). This differs from the AOSI Total Score, the summed score of all AOSI items and ranges from 0–38 (Bryson et al., 2008). While there are many studies exploring group differences using the AOSI in IL infant sibling populations, few studies directly report the scale's psychometric properties, or the data required to calculate them. This leads to challenges with evaluating what the optimal cut points are for the scale based on currently available evidence. Given that clinical measures should have cut points yielding sensitivity and specificity values exceeding 0.70 (Zwaigenbaum et al., 2015) and ideally between 0.80 and 0.90 if ascribing to Bayes Theorem (Medow & Lucey, 2011), determination of what cut point sensitivity and specificity thresholds are acceptable or even achievable given the cost of false positives and negatives should be considered when the AOSI is used in different IL infant contexts.

### Considerations for Future Data Collection and Analyses

First, when assessing the utility of a scale in a novel context, it is paramount to control for demographic factors that can confound results. For example, low SES is linked to poor outcomes in many areas of early development (Bradley & Corwyn, 2002; Chen et al., 2019; Freitas et al., 2013; Lawson et al., 2018) and can be affected by other related cofactors, such as ethnicity (Bradley & Corwyn, 2002). Papers included in this review may be biased due to a failure to control for the potential impacts of factors such as family SES and ethnicity. Finally, while ASD has been known to be related to advancing paternal age (Puleo et al., 2012), none of the studies in this review included it as a possible covariate. Future studies should include family demographics in their analysis to promote generalizability of findings.

Second, reliability and validity need to be reassessed in novel contexts**.** The presentation of ASD in FXS, TSC, DS, and infant siblings may manifest differently (Abbeduto et al., 2014). Thus, assessment of reliability and validity of ASD symptom assessment tools is warranted in novel IL populations. Reporting item-level data may aid in the identification of emergent patterns across IL populations (e.g., FXS infants with ASD have increased motor impairments relative to ASD infant siblings; Roberts et al., 2016).

Third, more stringent and explicitly stated inclusion and exclusion criteria are needed. Differences in exclusion criteria, for example, gestational age, birthweight, and the other neurological conditions, impact comparability and generalizability of results. Inclusion and exclusion criteria should be selected based on the study question. For example, preterm infants are at 3–4 times increased likelihood for ASD diagnosis relative to the general population (7% vs 0.76% respectively; Agrawal et al., 2018; Chen et al., 2006) and thus, should be considered a separate IL group. Premature infants also experience cognitive impairment that have a developmental interaction with SES (Tong et al., 2006; Torche & Echevarría, 2011).

Fourth, AOSI cut points (for the Total Score or number of Risk Markers) need to be reported. A paucity of literature addresses the AOSI’s prediction of ASD in FXS, TSC, and DS populations. When using the AOSI, it is imperative to describe explicitly how the measure was used, including cut points (both the actual cut point used and the metric [AOSI Total Score or Risk Markers]). Failure to do so can draw into question the validity of study results and undermine the generalizability of findings to other contexts.

Fifth, non-ASD or IL control groups are needed. Lack of appropriate LL control group(s) negates the possibility of investigating whether patterns of results are group or syndrome-specific (i.e., associated with IL status or ASD diagnosis) or reflect typical child development. Are the reported results which attempt to characterize ASD features specific to a particular IL population (e.g., infant siblings, FXS, TSC, DS) or is it possible that the reported findings are not specific to ASD or IL populations and instead are a feature of typical development? Future studies should include non-clinical comparison groups when using the AOSI with IL infant populations.

Sixth, it is important to consider age at outcome assessment. It is imperative when investigating early features of a condition like ASD that results are accurately attributed to the condition of interest. Diagnostic assessments at 24-months are less sensitive (Ozonoff et al., 2015). This is likely due to different groups of children being identified at 24- and 36-months (i.e., children diagnosed with ASD at 24-months generally have more severe symptom presentation than children diagnosed at 36-months; Zwaigenbaum et al. 2020). Since the goal of these studies is early detection, using 24-month outcome assessments (although likely to only capture a specific group of ASD children) is still pertinent.

Seventh, the age at which the AOSI is administered should be determined by the research question. AOSI Total Scores were not able to distinguish between IL and LL infants when administered at 6 and 9 months across the included studies. Given that meta-analyses report clear evidence of group differences emerging by 12-months of age and older among IL-ASD and LL or IL non-ASD infant populations, reliance of AOSI scores before 12-months for classification purposes is not recommended. If studies aimed to investigate the emergence of ASD symptoms across the developmental timespan from infancy to age at diagnosis, earlier AOSI administrations (at 6 and/or 9 months) could be warranted.

### Limitations

This is the first systematic review and meta-analysis evaluating the use and classification properties of the AOSI across IL infant populations. This review has several limitations. Though we conducted a thorough search for studies using the AOSI in IL infants in six databases, it is possible that we still may have missed some AOSI papers. In addition, although most studies identified using the AOSI were on IL infant siblings, few studies have applied the measure to FXS, TSC, DS, and other IL populations. Thirdly, we were unable to assess if different signs of ASD as measured by the AOSI manifested differently across IL populations due to a lack of availability of item-level reporting data from the published studies that were included in this systematic review. It is important to note, however, that several of the studies included in this review were the first to use the AOSI in their non-infant sibling IL cohort.

## Conclusion

This review summarized the results of research that assessed group differences and psychometric performance of the AOSI in populations of infants at IL for a diagnosis of ASD. Overall, group differences on the AOSI were consistently found by 12 months of age between IL-ASD and LL or IL non-ASD groups. However, we were not able to assess for differences in individual classification properties across different IL populations. As such, it is critical to investigate further the psychometric properties (i.e., sensitivity and specificity) of the AOSI across different IL populations in which phenotypic differences may exist. Ensuring study design and methodology are robust and transparent to not only protect against biasing factors, but also allow for comparison with similar or follow-up studies is important. Understanding the differences in methodology can inform future studies as researchers continue to investigate the early presentation of signs of ASD across diverse IL populations. Overall, the AOSI shows promise as an early detection tool for different infant groups at IL for ASD.

## Supplementary Information

Below is the link to the electronic supplementary material.

Supplementary file1 (DOCX 25.4 KB)

Supplementary file2 (DOCX 31 KB)

Supplementary file3 (DOCX 314 K)

## Data Availability

The data collected during this review that support the findings of this study are available from the corresponding author upon reasonable request.

## Abbreviations

**ASD**: Autism spectrum disorder
**CDC**: Centers for Disease Control and Prevention
**IL**: Increased likelihood
**FXS**: Fragile X Syndrome
**TSC**: Tuberous Sclerosis Complex
**AOSI**: Autism Observation Scale for Infants
**DS**: Down Syndrome
**PRISMA**: Preferred Reporting Items for Systematic Reviews and Meta Analyses
**LL**: Low likelihood
**SES**: Socioeconomic status
**ADOS**: Autism Diagnostic Observation Schedule
**ADI-R**: Autism Diagnostic Interview-Revised
**PDD**: Pervasive developmental disorder
**IL-ASD**: Infant siblings diagnosed with ASD
**CI**: Confidence interval
**Sibs-ASD**: Infant siblings diagnosed with ASD
**Sibs-N**: Infant siblings not diagnosed with ASD
**Sibs-DD**: Infant siblings who are developmentally delayed
**TSC-ASD**: Children with TSC who are diagnosed with ASD
