# Supplementary material for: Autism Observation Scale for Infants: Systematic Review and Meta-Analysis in Samples at Increased Likelihood of Autism Spectrum Disorders
Source: Rev J Autism Dev Disord. 2024 Jan 18;12(4):683–705. doi: 10.1007/s40489-023-00417-y (PMC12799723; doi:10.1007/s40489-023-00417-y)
Supplement: Supplementary file 1 — Supplementary file1 (DOCX 25.4 KB) [file 40489_2023_417_MOESM1_ESM.docx]

**Primary database searches**

**CINAHL Plus / ERIC Search via OVID – July 2022**

**Search as run:** ("autism observation scale for infants" OR "AOSI") AND ("autism" OR "autism spectrum disorders" OR "autistic disorder")

| **Databases being searched**: CINAHL Plus with Full Text, ERIC | | |
| --- | --- | --- |
|  | ("autism" OR "autism spectrum disorders" OR “autistic") | TX All Text |
| AND | ("autism observation scale for infants" OR "AOSI") | TX All Text |
| Limit: From January 2005 – July 2022 | | |

**Results**:

83 results found July 4^th^, 2022 (75 for CINAHL, 8 for ERIC). Search limited to publications between January 2005 and July 2022.

**Second Search July 26, 2023**

Filter: From 2022/7/ to 2023/7

Results: 3 results found July 26, 2023 (3 for CINAHL, 0 for ERIC); only 1 title/abstract mentioned using AOSI

**Total articles identified (both searches):** 78 from CINAHL, 8 from ERIC.

**JSTOR Search – July 2022**

**Search as run:**

| 1^st^ Keyword |  | “autism” | | All fields |
| --- | --- | --- | --- | --- |
| 2^nd^ Keyword | OR | “autism spectrum disorders” | | All fields |
| 3^rd^ Keyword | OR | “autistic disorder” | | All fields |
| 4^th^ Keyword | AND | “autism observation scale for infants” | | All fields |
| 5^th^ Keyword | OR | “AOSI” | | All fields |
| **Access type**: All content  **Item type**: n/a  **Language:** All languages  **Publication date**: From 2005/01/01 to 2022/07/04  **Journal or book title:** n/a  **ISBN:** n/a  **Journal filter**: n/a | | | | |
| **Date** | From: 2005/01/01 | | To: 2022/07/04 | |

**Results**:

74 results found July 4^th^, 2022. Search limited to publications between January 2005 and July 2022.

**Second Search July 26, 2023**

Filter: From 2022/7/05 to 2023/07/26

Results: 3 results found July 26, 2023; 0 mention autism or AOSI

**Total articles identified (both searches):** 77 from JSTOR.

**PubMed Search – July 2022**

**Search as run:**

((autism) OR (autism spectrum disorders) OR (autistic disorder)) AND ((autism observation scale for infants) OR (AOSI))

**Filter**: From 2005/1/1 to 2022/7/4

**Results:**

214 results found July 4^th^, 2022. Search limited to publications between January 2005 and July 2022.

**Second Search July 26, 2023**

Filter: From 2022/7/5/ to 2023/07/26

Results: 21 results found July 26, 2023; 1 one title/abstract mentioned using AOSI

**Total articles identified (both searches):** 235 from PubMed.

**Web of Science – July 2022**

**Search as run**:

|  | Topic | ("autism" OR "autism spectrum disorder" OR "autistic disorder") | | All fields |
| --- | --- | --- | --- | --- |
| AND | TOPIC | ("Autism Observation Scale for Infants" OR "AOSI") | | All fields |
| Index date | From: 2005-01-01 | | To: 2022-07-04 | |

**Results**

38 results found July 4^th^, 2022. Search limited to publications between January 2005 and July 2022.

**Second Search July 26, 2023**

Filter: From 2022/7/5/ to 2023/07/26

Results: 1 result found July 26, 2023; only 1 title/abstract mentioned using AOSI

**Total articles identified (both searches):** 39 from Web of Science.

**EMBASE/OVID – July 2022**

Search as run:

| **#** | **Searches** | **Results** |
| --- | --- | --- |
| 1 | Exp autism/ | 84582 |
| 2 | “autism spectrum disorder”.mp | 29729 |
| 3 | “autistic disorder” | 2618 |
| 4 | 1 or 2 or 3 | 87479 |
| 5 | “autism observation scale for infants”.mp | 36 |
| 6 | “AOSI”.mp | 60 |
| 7 | 5 or 6 | 75 |
| 8 | 4 and 7 | 44 |
| 9 | Limit 8 to yr=”2005 -Current” | 44 |

**Results**:

44 results found July 4^th^, 2022. Search limited to publications between January 2005 and July 2022.

**Second Search July 26, 2023**

Filter: Limit 8 to yr=”2022 – Current”

Results: 1 result found July 26, 2023; only 1 title/abstract mentioned using AOSI

**Total articles identified (both searches):** 35 from EMBASE.

**Grey literature searches**

**WorldCat.org – July 2022**

**Search as run:**

("autism" OR "autism spectrum disorders" OR “autistic disorder") AND ("autism observation scale for infants" OR "AOSI")

**Results:**

27 results found July 4^th^, 2022. Search limited to publications between January 2005 and July 2022.

**Second Search July 26, 2023**

Filter: 2022 – Current

Results: 3 result found July 26, 2023; only 1 title/abstract mentioned using AOSI

**Total articles identified (both searches):** 30 from WorldCat.org.

**Opengrey.eu – July 2022**

**Search as run:**

("autism" OR "autism spectrum disorders" OR “autistic disorder") AND ("autism observation scale for infants" OR "AOSI")

**Results:**

0 results found July 4^th^, 2022. No search limit was imposed as no data filter was accessible.

**Second Search July 26, 2023**

Filter: 2022 – Current

Results: 0 result found July 26, 2023

**Total articles identified (both searches):** 0 from Opengrey.eu.

**Greylit.org – July 2022**

**Search as run:**

((autism) OR (autism spectrum disorders) OR (autistic disorder)) AND ((autism observation scale for infants) OR (AOSI))

**Results**:

0 results found July 4^th^, 2022. Search limited to publications between January 2005 and July 2022.

**Second Search July 26, 2023**

Filter: 2022 – Current

Results: 0 result found July 26, 2023

**Total articles identified (both searches):** 0 from Greylit.org.
