# Supplementary material for: Autism Observation Scale for Infants: Systematic Review and Meta-Analysis in Samples at Increased Likelihood of Autism Spectrum Disorders
Source: Rev J Autism Dev Disord. 2024 Jan 18;12(4):683–705. doi: 10.1007/s40489-023-00417-y (PMC12799723; doi:10.1007/s40489-023-00417-y)
Supplement: Supplementary file 3 — Supplementary file3 (DOCX 314 K) [file 40489_2023_417_MOESM3_ESM.docx]

**RESULTS**

**Confirmation of ASD in Proband of Infant Siblings**

IL infant sibling status varied across the 13 studies that had IL infant sibling study participants: [Zwaigenbaum et al., 2005](https://doi.org/10.1016/j.ijdevneu.2004.05.001), [Zwaigenbaum et al., 2020](https://doi.org/10.1111/cdev.13485), [Zwaigenbaum et al., 2021](https://doi.org/10.1111/jcpp.13417), and [Sacrey et al., 2018](https://doi.org/10.1002/aur.1920) required confirmation of the older sibling’s ASD diagnosis via clinical assessment or review of diagnostic records using DSM criteria, [Estes et al., 2015](https://doi.org/10.1186/s11689-015-9117-6) required older siblings to meet criteria on the Social Communication Questionnaire (SCQ) and ADI-R, [Gammer et al., 2015](https://doi.org/10.1016/j.infbeh.2014.12.017), [Gilga et al., 2015](http://dx.doi.org/10.1016/j.cub.2015.05.011), [Bedford et al., 2017](https://doi.org/10.1186/s13229-017-0167-3), and [Bedford et al., 2019](https://doi.org/10.1186/s11689-019-9274-0) confirmed the community clinical diagnosis of ASD using the Development and Well Being Assessment (DAWBA) and SCQ, and [Hahn et al., 2017](https://doi.org/10.1016/j.ridd.2017.10.004) via documentation of an ASD diagnosis (though what this entails was not specified). Though [Bussu et al., 2018](https://doi.org/10.1007/s10803-018-3509-x), [Roberts et al., 2016](https://doi.org/10.1007/s10803-016-2903-5), and [Bedford et al., 2016](https://doi.org/10.1186/s13229-016-0081-0) included IL infant siblings participants, they did not specify how the older sibling’s diagnosis of ASD was confirmed by study authors.

**Inclusion criteria**

Explicit inclusion criteria for participants were detailed for all seventeen studies but varied according to the goals and objectives of each study. For instance, of the 13 studies that included infant sibling participants, designation as an infant sibling was the most stringent for [Zwaigenbaum et al., 2005](https://doi.org/10.1016/j.ijdevneu.2004.05.001), [Zwaigenbaum et al., 2020](https://doi.org/10.1111/cdev.13485), [Zwaigenbaum et al., 2021](https://doi.org/10.1111/jcpp.13417), and [Sacrey et al., 2018](https://doi.org/10.1002/aur.1920) due to their required confirmation of the older sibling's ASD diagnosis through either clinical assessment or review of diagnostic records using DSM-IV-TR criteria. All other studies with infant sibling participants either required the older sibling to meet criteria for ASD on some ASD measure (SCQ, ADI-R, DAWBA) or made no mention of how the older sibling received an ASD diagnosis. For the two studies with FXS infants ([Hahn et al., 2017](https://doi.org/10.1016/j.ridd.2017.10.004), [Roberts et al., 2016](https://doi.org/10.1007/s10803-016-2903-5)), infant status with FXS required confirmed by genetic report. For the three studies with TSC infants, infant TSC status was based on clinical presentation or genetic workups ([McDonald et al., 2017](https://doi.org/10.1002/aur.1846)), meeting clinical or genetic criteria for TSC ([Capal et al., 2017](https://doi.org/10.1016/j.pediatrneurol.2017.06.010)), or via being recruited from TSC specialty clinics, newborn nurseries, pediatrician offices, or met genetic criteria for TSC diagnosis ([Jeste et al., 2014](https://doi.org/10.1212/WNL.0000000000000568)). For the one study of infants with DS, while no confirmatory testing was done by study authors, infants were recruited from three other pilot studies examining the infant neurogenetic syndromes who themselves recruited participants based on flyers with local parent groups, DS clinics, and ongoing research studies in the United States [(](https://www.ncbi.nlm.nih.gov/pmc/articles/PMC7357991/)[Hahn et al., 2020](https://pubmed.ncbi.nlm.nih.gov/32661519)). Overall, none of the studies that recruited infants with FXS, TSC, or DS described how infant IL status was confirmed relative to any DSM or ICD criterion.

Inclusion criteria for LL controls varied considerably across the 15 studies which employed them. LL inclusion criteria ranged from explicitly detailed and descriptive to increasingly sparse and lacking detail. [Gilga et al., 2015](http://dx.doi.org/10.1016/j.cub.2015.05.011) had the most robust inclusion criteria for LL controls; they required (A) control infants to have an older sibling who was born full-term with a normal birth weight, and (B) control infants to lack ASD diagnoses in any first-degree family members confirmed by either parent interview or family medical history. Similarly, [Estes et al., 2015](https://doi.org/10.1186/s11689-015-9117-6) required LL control infants to (A) have an older sibling who did not meet criteria SCQ or Family Interview for Genetic Studies (FIGS) criteria for ASD, and (B) a lack of first-degree relatives with ASD or intellectual disability. [Roberts et al., 2016](https://doi.org/10.1007/s10803-016-2903-5) was less descriptive and simply required an absence of suspected delays and no familial history or indicator of ASD in LL controls. [Gammer et al., 2015](https://doi.org/10.1016/j.infbeh.2014.12.017) required LL controls to (A) not have first degree relatives with ASD, and (B) have an older sibling (full or half) that did not meet criteria for ASD on the SCQ (aka did not meet the cut-off of ≥ 15 on the SCQ). [Bedford et al., 2017](https://doi.org/10.1186/s13229-017-0167-3) and [Bedford et al., 2019](https://doi.org/10.1186/s11689-019-9274-0) both had similar inclusion criteria, but only required LL controls to have an older sibling that did not meet criteria for ASD on the SCQ (did not score ≥ 15). [Zwaigenbaum et al., 2005](https://doi.org/10.1016/j.ijdevneu.2004.05.001), [Zwaigenbaum et al., 2020](https://doi.org/10.1111/cdev.13485), and [Zwaigenbaum et al., 2021](https://doi.org/10.1111/jcpp.13417) all required LL controls to lack a first- or second-degree relative with an ASD diagnosis. [Bussu et al., 2018](https://doi.org/10.1007/s10803-018-3509-x) had the least restrictive LL control inclusion criteria and only required control infants to have an older full sibling with typical development. While [Jeste et al., 2014](https://doi.org/10.1212/WNL.0000000000000568) and [Hahn et al., 2020](https://pubmed.ncbi.nlm.nih.gov/32661519) both recruited LL control infants from either IRB-approved infant databases or from other studies respectively. Three studies ([McDonald et al., 2017](https://doi.org/10.1002/aur.1846), [Hahn et al., 2017](https://doi.org/10.1016/j.ridd.2017.10.004), [Bedford et al., 2016](https://doi.org/10.1186/s13229-016-0081-0)) did not specify how LL controls were recruited or the criterion used to do so. Only two studies ([Capal et al., 2017](https://doi.org/10.1016/j.pediatrneurol.2017.06.010), [Sacrey et al., 2018](https://doi.org/10.1002/aur.1920)) did not employ LL control groups .

**Exclusion criteria**

Explicit exclusion criteria for IL participants were detailed for only 11 studies and varied according to their respective goals and objectives. [Estes et al., 2015](https://doi.org/10.1186/s11689-015-9117-6) excluded participants if they had genetic conditions or syndromes, sensory impairments (e.g., vision or hearing loss), had a birth weight <2,000g, gestational age of <36 weeks at birth, or suffered significant perinatal adversity and/or were exposed *in utero* to neurotoxins, had any MRI contraindications, had a predominant household language that was not English, were adopted or half-siblings, had a first degree relative with psychosis, schizophrenia, or bipolar disorder, or were twins. Likewise, [Capal et al., 2017](https://doi.org/10.1016/j.pediatrneurol.2017.06.010) excluded IL infants if they were born preterm (<36 weeks), suffered significant perinatal complications, were administered investigational drugs as part of other research studies, were taking an mTOR inhibitor at the time of enrollment, had Subependymal giant Cell Astrocytoma necessitating medical/surgical treatment, had a history of epilepsy, or had any MRI contraindications. The remaining 9 studies employed exclusion criteria of varying detail and robustness. [Zwaigenbaum et al., 2020](https://doi.org/10.1111/cdev.13485), [Sacrey et al., 2018](https://doi.org/10.1002/aur.1920), and [Zwaigenbaum et al., 2021](https://doi.org/10.1111/jcpp.13417) all broadly excluded IL participants if they were not born full-term, had a birth weight <2,500g, and/or had significant neurologic, genetic, or sensory-motor conditions. While similar, [Zwaigenbaum et al., 2005](https://doi.org/10.1016/j.ijdevneu.2004.05.001) only excluded participants if they did not have term gestation or had a birth weight <2,500g. [Roberts et al., 2016](https://doi.org/10.1007/s10803-016-2903-5) and [Gammer et al., 2015](https://doi.org/10.1016/j.infbeh.2014.12.017) excluded IL participants if they were not born full-term and had significant neurological or developmental conditions. [Gilga et al., 2015](http://dx.doi.org/10.1016/j.cub.2015.05.011) and [Bedford et al., 2016](https://doi.org/10.1186/s13229-016-0081-0) both excluded IL participants if they had significant medical or developmental conditions. [Bussu et al., 2018](https://doi.org/10.1007/s10803-018-3509-x) excluded participants who lacked 36-month clinical ASD outcome evaluation. The remaining 6 studies [(](https://www.ncbi.nlm.nih.gov/pmc/articles/PMC7357991/)[Hahn et al., 2020](https://pubmed.ncbi.nlm.nih.gov/32661519), [McDonald et al., 2017](https://doi.org/10.1002/aur.1846), [Hahn et al., 2017](https://doi.org/10.1016/j.ridd.2017.10.004), [Bedford et al., 2017](https://doi.org/10.1186/s13229-017-0167-3), [Bedford et al., 2019](https://doi.org/10.1186/s11689-019-9274-0), [Jeste et al., 2014](https://doi.org/10.1212/WNL.0000000000000568)) did not detail exclusion criteria for IL participants.

Exclusion criteria for LL controls were explicitly detailed for only 10 studies and varied considerably. [Jeste et al., 2014](https://doi.org/10.1212/WNL.0000000000000568) and [McDonald et al., 2017](https://doi.org/10.1002/aur.1846) both excluded LL controls if they were born preterm (<37 weeks of gestation), suffered birth trauma, had developmental concerns, or had any family history of ASD or intellectual disability. [Roberts et al., 2016](https://doi.org/10.1007/s10803-016-2903-5) excluded LL controls if they were not born full-term, had significant neurological or developmental conditions, and/or if they had developmental composite scores >1 standard deviation away from the mean. The exclusion criteria for LL controls was identical to that of IL participants for [Zwaigenbaum et al., 2005](https://doi.org/10.1016/j.ijdevneu.2004.05.001), [Gammer et al., 2015](https://doi.org/10.1016/j.infbeh.2014.12.017), [Estes et al., 2015](https://doi.org/10.1186/s11689-015-9117-6), [Bussu et al., 2018](https://doi.org/10.1007/s10803-018-3509-x), [Zwaigenbaum et al., 2020](https://doi.org/10.1111/cdev.13485), [Bedford et al., 2016](https://doi.org/10.1186/s13229-016-0081-0), and [Zwaigenbaum et al., 2021](https://doi.org/10.1111/jcpp.13417) as described in the paragraph above. Five studies did not report LL control exclusion criteria ([Hahn et al., 2020](https://pubmed.ncbi.nlm.nih.gov/32661519), [Hahn et al., 2017](https://doi.org/10.1016/j.ridd.2017.10.004), [Hahn et al., 2017](https://doi.org/10.1016/j.ridd.2017.10.004), [Bedford et al., 2017](https://doi.org/10.1186/s13229-017-0167-3), [Bedford et al., 2019](https://doi.org/10.1186/s11689-019-9274-0)) while two ([Capal et al., 2017](https://doi.org/10.1016/j.pediatrneurol.2017.06.010), [Sacrey et al., 2018](https://doi.org/10.1002/aur.1920)) did not employ LL control comparison groups in their study design.

**Study design and interrater reliability**

Though 15 studies were longitudinal and 2 cross-sectional, each administered the AOSI at a single or multiple timepoints (ranging between 6, 9, 12, 15, or 18 months) and either (A) compared AOSI scores against a later ASD classification at 24-months or diagnostic assessment at 36-months, (B) compared AOSI scores across IL/LL study groups, (C) compared AOSI scores against scores on other early measures of autism symptoms, or (D) used AOSI scores in various statistical models (logistical regression, trajectory analysis, mixed modelling, multilevel modelling, autoregression, and machine learning). AOSI Total Scores, Risk Markers, or item-level data were analyzed by group membership (infant siblings, FXS, TSC, DS) against 24-month, 36-month, or 7-year ASD outcomes.

Though AOSI reliability data has been previously reported [Bryson et al., 2008](https://doi.org/10.1007/s10803-007-0440-y), AOSI reliability assessments were conducted by 6 of studies included in this review ([Zwaigenbaum et al., 2005](https://doi.org/10.1016/j.ijdevneu.2004.05.001), [Hahn et al., 2017](https://doi.org/10.1016/j.ridd.2017.10.004), [Roberts et al., 2016](https://doi.org/10.1007/s10803-016-2903-5), [Bedford et al., 2016](https://doi.org/10.1186/s13229-016-0081-0), [Bedford et al., 2017](https://doi.org/10.1186/s13229-017-0167-3), [Bedford et al., 2019](https://doi.org/10.1186/s11689-019-9274-0)). [Zwaigenbaum et al., 2005](https://doi.org/10.1016/j.ijdevneu.2004.05.001) reports three main reliability estimates: (1) absolute agreement between raters of >90% for each AOSI item, (2) interrater agreement of the AOSI total score of 0.71, 0.90, and 0.92 for 6-, 12-, and 18-month AOSI administrations, and (3) a test-retest reliability at 12-month AOSI administrations of 0.63. [Roberts et al., 2016](https://doi.org/10.1007/s10803-016-2903-5) and [Hahn et al., 2017](https://doi.org/10.1016/j.ridd.2017.10.004) both double-coded 20% of AOSI assessments and report an item-level inter-rater reliability of 0.89. While [Bedford et al., 2016](https://doi.org/10.1186/s13229-016-0081-0), [Bedford et al., 2017](https://doi.org/10.1186/s13229-017-0167-3), and [Bedford et al., 2019](https://doi.org/10.1186/s11689-019-9274-0) all report double-coding the majority of AOSI assessments and report an intraclass correlation coefficient of 0.95, these studies all focus on the same sample of IL and LL infants and should be considered as one reliability estimate, not three.

**Meta-analyses**

**IL-developmentally delayed (IL-DD) and IL-ASD**

**Between 6 and 10 months**. A total of three comparisons of AOSI Total Scores were included in this meta-analysis. There was no effect of AOSI Total Score, suggesting that the IL-ASD group did not differ from the IL-DD control group (Cohen’s *d*= 0.16, 95% CI = -0.09 - 0.41, *z* = 1.25, *p* = 0.21, Supplemental Figure 1a).

**Between 12 and 14 months**. A total of two comparisons of AOSI Total Scores were included in the meta-analysis. There was no effect of AOSI Total Score, suggesting that the IL-ASD group did not differ from IL-DD for AOSI Total Scores (Cohen’s *d* = 0.21, 95% CI = -0.05 - 0.47, *z* = 1.61, *p* = 0.11, Supplemental Figure 1b)**.**

| 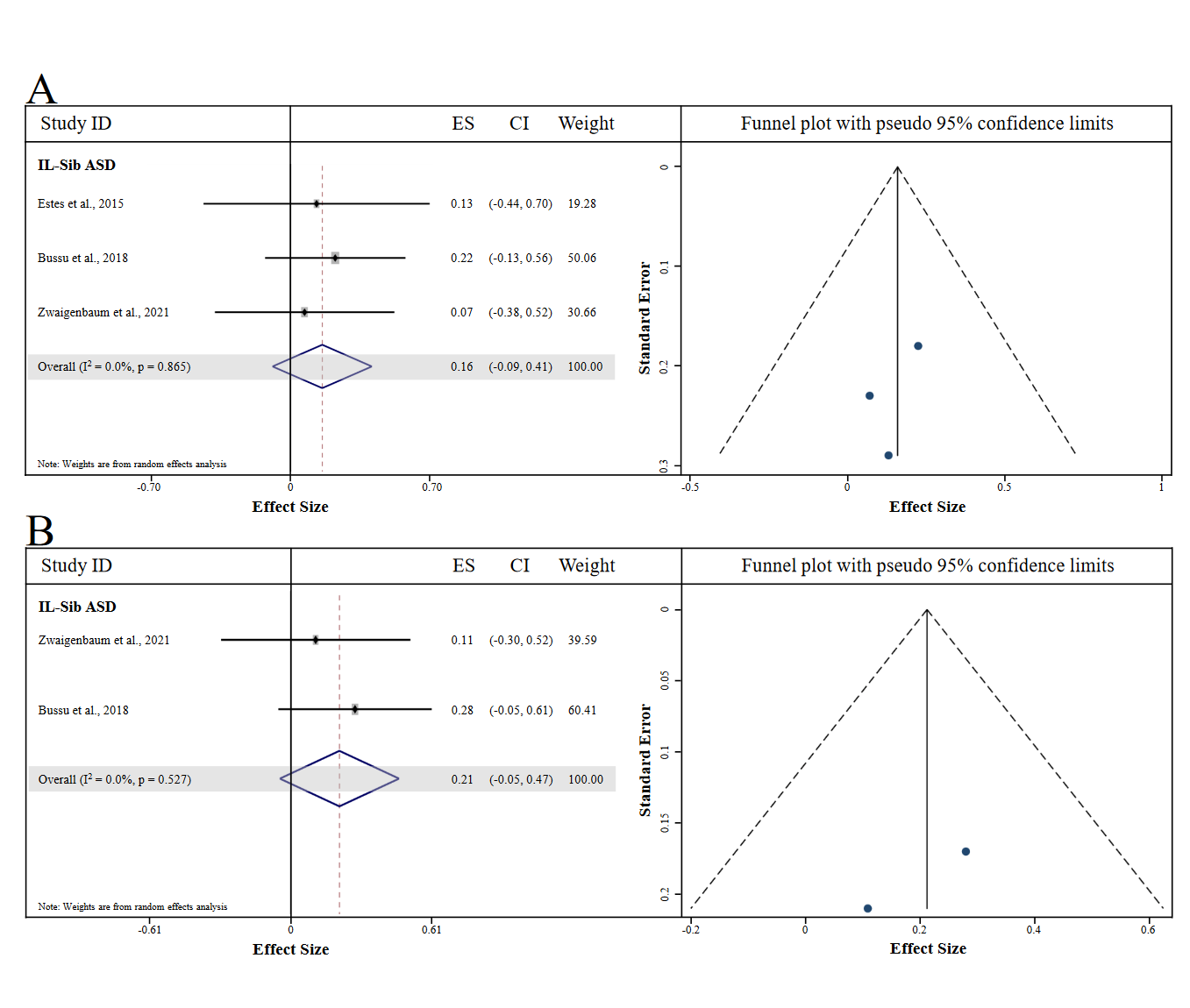 |
| --- |
| **Supplementary Figure S1 a, b \|** Meta-Analysis comparing IL-developmentally delayed (IL-DD) and IL-ASD (left) with the Trim and Fill Plot (right). A = for ages 6–10 months, B = for ages 12–14 months |

**IL-infants with typical development vs IL-ASD**

**Between 6 and 10 months**. A total of three comparisons of AOSI Total Scores were included in this meta-analysis. There was an effect of AOSI Total Score, suggesting that the IL-ASD group differ from the IL-typical control group (Cohen’s *d*= 0.29, 95% CI = 0.10 - 0.49, *z* = 2.91, *p* = 0.004, Supplemental Figure 2a).

**Between 12 and 14 months**. A total of two comparisons of AOSI Total Scores were included in the meta-analysis. There was an effect of AOSI Total Score, suggesting that the IL-ASD group differed from IL-typical for AOSI Total Scores (Cohen’s *d* = 0.74, 95% CI = 0.51 - 0.97, *z* = 6.35, *p* < 0.001, Supplemental Figure 2b)**.**

| 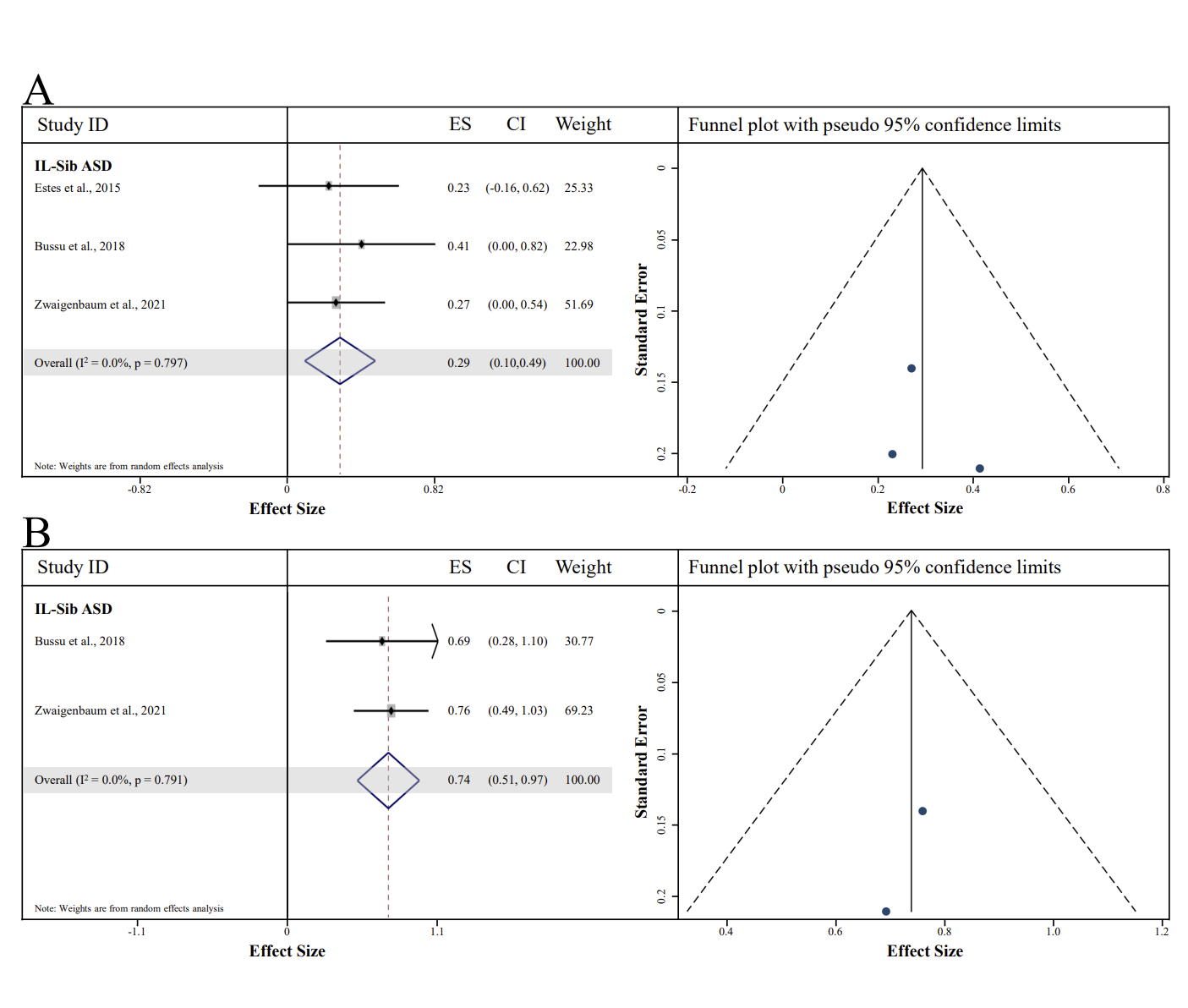 |
| --- |
| **Supplementary Figure S2 a, b \|** Meta-Analysis comparing IL-infants with typical development vs IL-ASD (left) with the Trim and Fill Plot (right). A = for ages 6–10 months, B = for ages 12–14 months |
